# PERM1 Gene Therapy Prevents Pressure Overload-induced Heart Failure Through a Sarcomere-Mitochondria Energetic Microdomain

**DOI:** 10.21203/rs.3.rs-10832916/v1

**Published:** 2026-09-14

**Authors:** Karthi Sreedevi, Abigail O. Doku, Alexey V. Zaitsev, Ryan N. Montalvo, Clare L. Dennison, Audrey Korte, Sarah Salama, Mysha Alabbi, Samia Tasneem, Rebekah Thomas, Mark C. Renton, Seby Edassery, Stephanie M. Deditz, Steven Burrows, Garima Patel, Scott R. Johnstone, Zhen Yan, James W. Smyth, Jonathan A. Kirk, Junco S. Warren

**Affiliations:** 1 Fralin Biomedical Research Institute at Virginia Tech Carilion, Virginia Tech, Roanoke, VA, USA; 2 Center for Vascular and Heart Research, Fralin Biomedical Research Institute, Virginia Tech, Roanoke, VA, USA; 3 Translational Biology Medicine and Health Graduate Program, Virginia Tech, Blacksburg, VA, USA; 4 Center for Exercise Medicine, Fralin Biomedical Research Institute, Virginia Tech, Roanoke, VA, USA; 5 Tissue Processing and Electron Microscopy Core, Fralin Biomedical Research Institute, Virginia Tech, Roanoke, VA, USA; 6 Virginia Tech Carilion School of Medicine, Virginia Tech, Roanoke, VA, USA; 7 Department of Biological Sciences, College of Science, Virginia Tech, Blacksburg, VA, USA; 8 Department of Cell and Molecular Physiology, Loyola University Chicago, Chicago, Illinois, USA; 9 Department of Medicine, Section of Cardiology, University of Chicago, IL, USA; 10 Metabolomics Core, Fralin Biomedical Research Institute, Virginia Tech, Roanoke, VA, USA; 11 Department of Human Nutrition, Food and Exercise, Virginia Tech, Blacksburg, VA, USA

**Keywords:** Heart failure, HFrEF, AAV gene therapy, PERM1, Mitochondrial bioenergetics, Mechano-energetic coupling, Mitochondrial protein homeostasis, O-GlcNAcylation

## Abstract

Heart failure with reduced ejection fraction (HFrEF) remains a major cause of morbidity and mortality worldwide, characterized by impaired contractile function and mitochondrial dysfunction, yet therapies that simultaneously restore cardiac energetics and mechanical performance remain limited. Here, we show that adeno-associated virus-mediated overexpression of PERM1 (AAV-PERM1), a striated muscle-specific mitochondrial regulator, prevents pressure overload-induced HFrEF through a non-transcriptional mechanism that preserves both mitochondrial function and contractility. AAV-PERM1 mitigated declines in mitochondrial respiration and mitochondrial DNA content, maintained mitochondrial morphology under pressure overload, and attenuated pathological hypertrophy and fibrosis. Unexpectedly, these cardioprotective effects occurred despite persistent suppression of oxidative phosphorylation and fatty acid oxidation transcripts. Instead, PERM1 localized to a mitochondria–sarcomere microdomain, where it associated with ribosomal proteins and a creatine kinase-troponin C complex, suppressed pathological O-GlcNAcylation, and post-transcriptionally preserved electron transport chain protein abundance. Functionally, PERM1 enhanced myofibrillar force generation. These findings identify a previously unrecognized mechanism by which PERM1 preserves mitochondrial protein homeostasis and couples energy production to force generation, establishing a new paradigm for post-transcriptional metabolic control and positioning PERM1 as a therapeutic target for heart failure.

## 1. Introduction

Heart failure with reduced ejection fraction (HFrEF) is a life-threatening disease affecting about 27 million people worldwide and approximately 3 million people in the United States. The current treatment of HFrEF primarily focuses on neurohormonal antagonists, β-blockers, angiotensin-converting enzyme inhibitors, and angiotensin receptor blockers. More recently, sodium-glucose cotransporter 2 inhibitors, which was originally prescribed for type 2 diabetes, have been FDA-approved for patients of both HFrEF and heart failure with preserved ejection fraction. While these therapies have improved patient outcomes, high mortality and hospitalization rates persist.

Emerging therapeutic strategies for HFrEF increasingly aim to directly improve the underlying defects in the heart rather than merely managing symptoms^1^. Numerous studies have demonstrated that mitochondrial impairment and metabolic dysfunction render the heart more susceptible to pathological stress and contribute to the progression of HFrEF^2–4^. Over the past two decades, intensive studies have identified the transcription factors that orchestrate the expression of genes involved in mitochondrial bioenergetics in response to various physiological and pathophysiological stimuli^3–7^. Among these, peroxisome proliferator-activated receptor gamma coactivator-1 alpha (PGC-1α) is a central regulator of mitochondrial biogenesis and oxidative metabolism, and the maintenance of PGC-1α signaling has been proposed as a potential therapeutic strategy to ameliorate mitochondrial impairment and contractile dysfunction in heart failure^2^. However, studies have shown that transgenic mice overexpressing PGC-1α develop cardiomyopathy due to uncontrolled mitochondrial biogenesis, which disrupts sarcomere integrity and contractile function^8^. In addition, moderate overexpression of PGC-1α (~3-fold) in mouse hearts accelerated the decline of systolic function and increased mortality during pressure overload despite enhanced mitochondrial gene expression and citrate synthase activity^9^. Similarly, overexpression and activation of key transcription factors regulating mitochondrial function and fatty acid metabolism, such as estrogen-related receptor (ERR) gamma and peroxisome proliferator-activated receptor (PPAR) in mice worsened cardiac dysfunction and reduced survival during pressure overload^10,11^. These studies suggest that enhancing mitochondrial biogenesis alone is insufficient and highlight the need for mechanisms that coordinate mitochondrial adaptation with contractile function during pathological stress.

PERM1 (PPARGC1- and ESRR-induced regulator in muscle 1) is a striated muscle-enriched regulator of mitochondrial bioenergetics^12^. We previously showed that PERM1 expression is reduced in both patients with HFrEF and mice subjected to pressure overload, whereas *Perm1*-KO mice exhibit reduced contractile performance and cardiac energy reserve accompanied by global downregulation of proteins involved in oxidative phosphorylation (OXPHOS). Conversely, PERM1 overexpression via adeno-associated virus (AAV) enhances systolic function, mitochondrial biogenesis, and exercise capacity in mice^13^. Furthermore, our recent study showed that PERM1 expression is fully restored in patients who exhibit myocardial recovery following left ventricular assist device implantation^14^, suggesting a potential link between PERM1 expression and functional recovery. However, whether restoration of PERM1 is sufficient to preserve cardiac function during pathological stress, and the mechanisms through which PERM1 coordinates mitochondrial energetics with contractile performance, remain unknown. Here, we tested the hypothesis that PERM1 coordinates mitochondrial energetics with contractile function during pathological stress. Using AAV9-mediated PERM1 delivery in a mouse model of pressure overload-induced HFrEF, we demonstrate that AAV-PERM1 prevents the development of HFrEF by preserving both myocardial contractile function and mitochondrial bioenergetic capacity during pathological stress. Mechanistically, we reveal that PERM1 maintains cardiac energetics through previously unrecognized post-transcriptional mechanisms that enhance mitochondrial biogenesis and protein homeostasis, allowing the stressed heart to sustain energetic-mechanical coupling despite pathological stress. This study further identifies AAV-PERM1 as a promising therapeutic strategy to prevent HFrEF.

## 2. Results

### AAV-PERM1 prevents systolic dysfunction and pathological remodeling during pressure overload.

**Figure 1.**
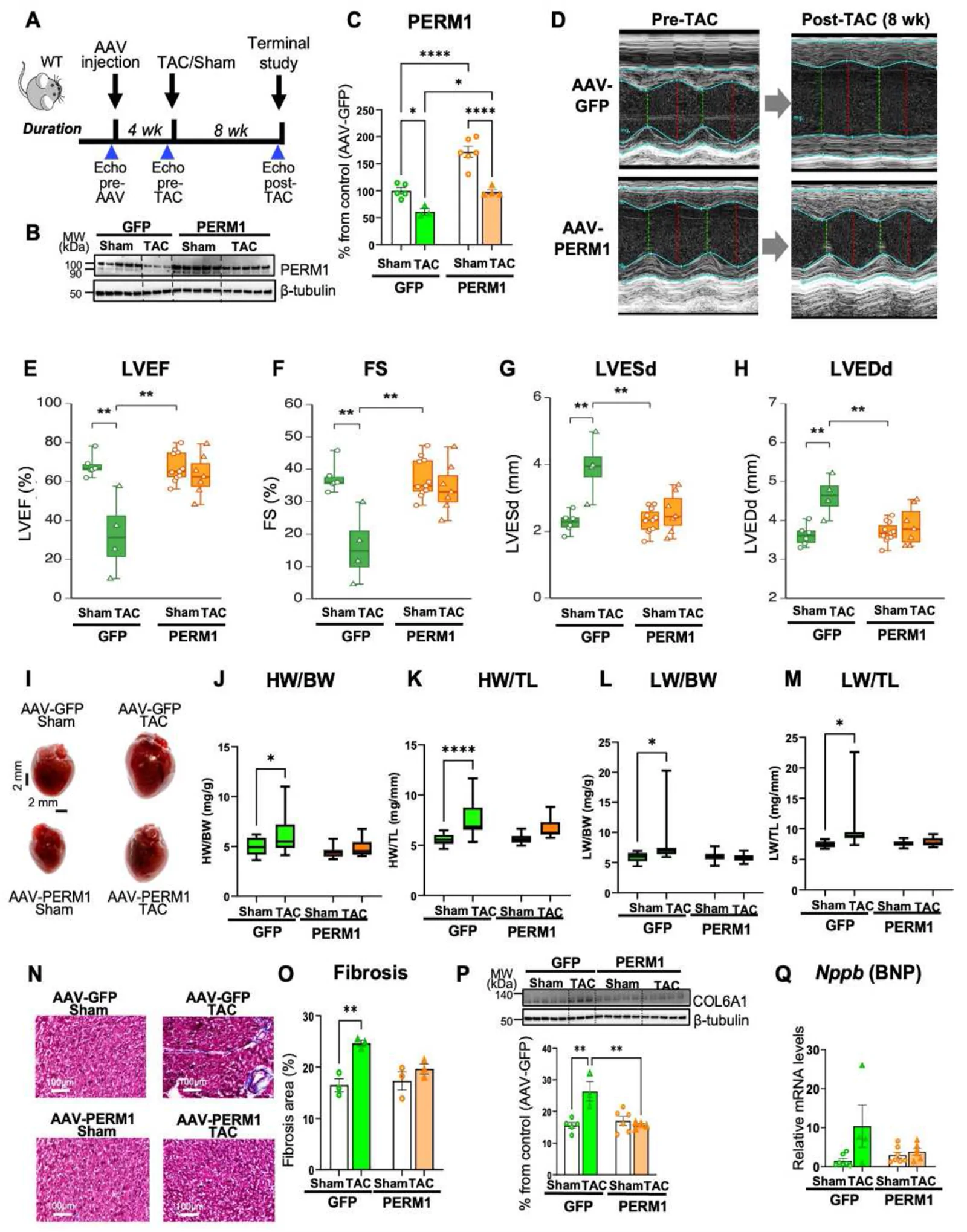
AAV-PERM1 prevents the major manifestations of heart failure during pressure overload in mice. **A,** Experimental design of the preventive study using AAV-PERM1 in mice. Blue triangles indicate the time points at which echocardiography was performed. **B-C,** Western blot analysis of cardiac tissue showing that TAC-induced downregulation of PERM1 was normalized by AAV-PERM1. **D-H,** Left ventricular ejection fraction (LVEF), fractional shortening (FS), left ventricular end-systolic diameter (LVESd), and left ventricular end-diastolic diameter (LVEDd) at 8 weeks post-TAC, along with representative M-mode echocardiographic images for each group. **I,** Representative gross images of AAV-GFP and AAV-PERM1-treated hearts in sham and TAC. **J,** Heart weight-to-body weight (HW/BW) ratio. **K,** Heart weight-to-tibial length (HW/TL) ratio. **L,** Lung weight-to-body weight (LW/BW) ratio. **M,** Lung weight-to-tibial length (LW/TL) ratio. **N-O,** Masson’s trichrome staining of cardiac tissue. **P,** Western blot analysis showing the expression of COL6A1, an extracellular matrix protein with cardiac fibrosis remodeling. **Q,** qPCR analysis of cardiac tissue showing the expression of *Nppb* (BNP), a marker of cardiac stress. Data are presented as mean ± SEM.*: p<0.05, **: p<0.01, ***: p<0.001, ****p<0.0001. Statistical analyses were performed using two-way ANOVA followed by Šidák post hoc pairwise comparisons test, except for panels **E-H**, which were analyzed in Python as described in the Methods.

To determine whether restoration of PERM1 expression protects against the development of HFrEF, transverse aortic constriction (TAC) surgery was performed in C57BL/6 mice four weeks after retro-orbital administration of either AAV9-GFP (control) or AAV9-PERM1 (Fig. 1A). Hearts were harvested 8 weeks after TAC/Sham (12 weeks after AAV treatment) (Fig. 1A). Consistent with our previous study^15^, TAC markedly reduced endogenous PERM1 expression, whereas AAV-PERM1 restored PERM1 abundance to levels comparable to sham controls (Fig. 1B–C). Pressure overload induced severe systolic dysfunction in AAV-GFP mice, with left ventricular ejection fraction (LVEF) declining from 68% to 32% (Fig. 1D–E, **Table S1**). In contrast, AAV-PERM1 completely preserved systolic function, maintaining LVEF and fractional shortening at levels indistinguishable from sham-operated mice throughout 8-week period (Fig. 1D–F, **Table S1**). AAV-PERM1 also prevented ventricular dilation, as evidenced by preservation of left ventricular end systolic diameter (LVESd) and end diastolic diameter (LVEDd) (Fig. 1G–H, **Table S1**).

In addition to preserving systolic function, AAV-PERM1 attenuated pathological remodeling. AAV-PERM1 abolished TAC-induced cardiac hypertrophy and pulmonary congestion, as manifested by normalization of the heart weight-to-body weight (HW/BW), heart weight-to-tibial length (HW/TL), lung weight-to-body weight (LW/BW), and lung weight-to-tibial length (LW/TL) ratios (Fig. 1I–M). Masson’s trichrome staining of cardiac tissue showed that AAV-PERM1 suppressed TAC-induced interstitial fibrosis (Fig. 1N–O). Consistently, the elevated expression of COL6A1, an extracellular matrix protein associated with cardiac fibrotic remodeling, observed in GFP-TAC hearts was absent in PERM1-TAC hearts (Fig. 1P). Moreover, the robust induction of the cardiac stress marker *Nppb* following TAC was prevented by AAV-PERM1 (Fig. 1Q). To confirm the cardiac-specific activity of the vector, AAV-PERM1 was administered to *Perm1*-KO mice. AAV-PERM1 restored cardiac PERM1 expression, with predominant expression of the 100-kDa isoform, and significantly improved LVEF compared with AAV-GFP-treated KO mice (**Fig. S1**).

In summary, these results demonstrate that restoration of PERM1 expression by AAV gene transfer prevents the major hallmarks of pressure overload-induced heart failure, including systolic dysfunction, pathological hypertrophy, and fibrosis.

### PERM1 preserves mitochondrial respiration capacity and limits electron leak during pressure overload.

**Figure 2.**
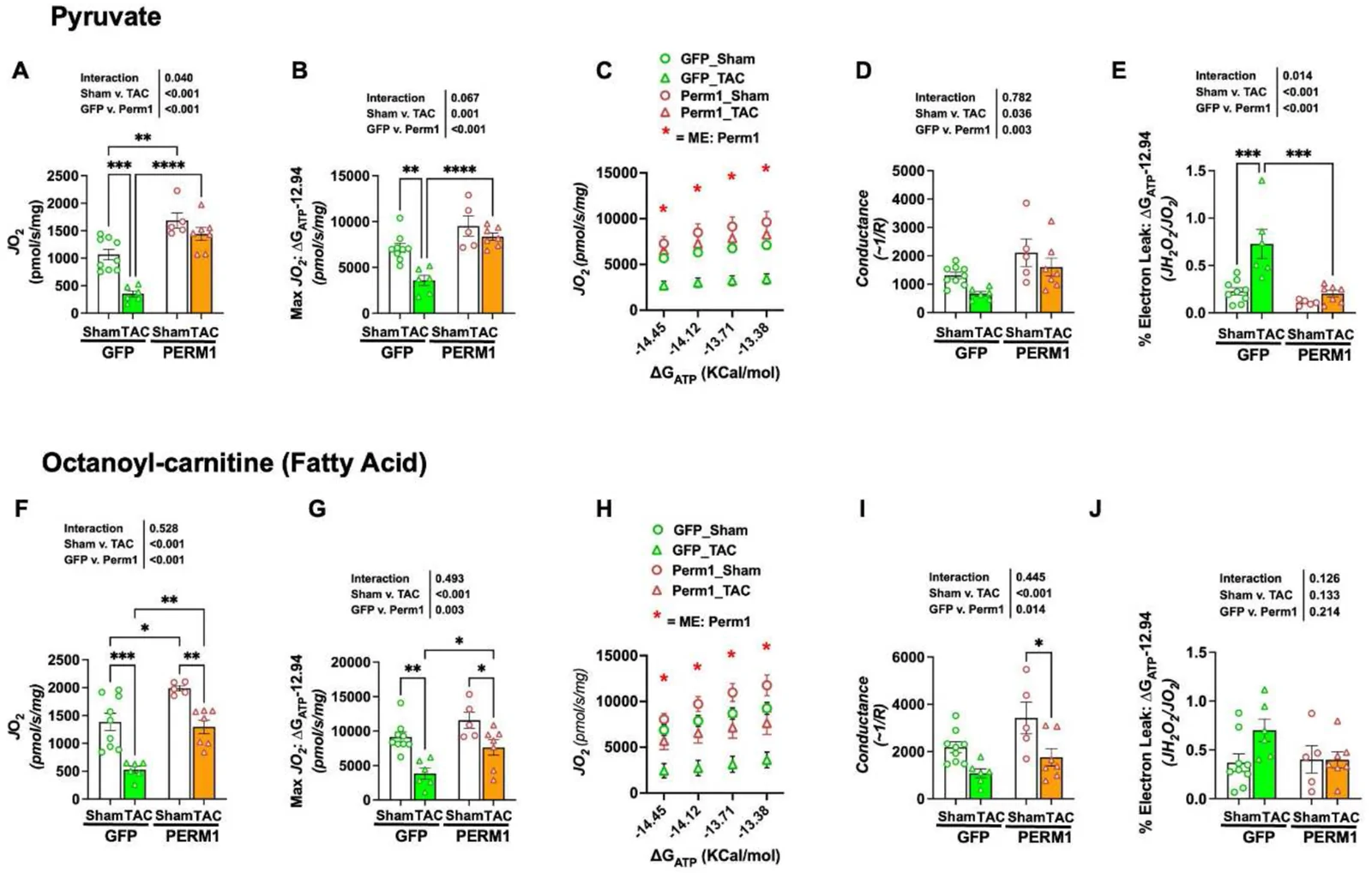
AAV-PERM1 preserves mitochondrial respiratory capacity and reduces electron leak during pressure overload. Mitochondria were isolated from hearts of AAV-GFP- and AAV-PERM1-treated mice 8 weeks after sham or TAC surgery. Pyruvate/malate and octanoyl-carnitine/malate were used as mitochondrial substrates to assess carbohydrate- and fatty acid-supported respiration, respectively. **A-C; F-H**, Non-phosphorylating oxygen consumption rate (*J*O_2_), maximal *J*O_2_, and *J*O_2_ plotted as a function of ATP free energy (ΔG_ATP_). **D; I**, Mitochondrial conductance derived from the linear relationship between *J*O_2_ and ΔG_ATP_. **E; J**, Electron leak, expressed as the percentage of *J*H_2_O_2_ relative to *J*O_2_ (%*J*H_2_O_2_/JO_2_). Data are presented as mean ± SEM. *: p<0.05, **: p<0.01, ***: p<0.001, ****p<0.0001. Statistical analyses were performed using two-way ANOVA followed by Šidák post hoc pairwise comparisons test.

To determine whether the cardioprotective effects of AAV-PERM1 during pressure overload were associated with preserved mitochondrial energetics, we measured mitochondrial respiration in isolated cardiac mitochondria using the Oroboros O2k system with a creatine kinase clamp^16^. Because fatty acid and glucose-derived pyruvate are the principal mitochondrial energy-producing pathways in the heart, mitochondrial respiratory capacity supported by octanoyl-carnitine/malate or pyruvate/malate, respectively, was assessed to determine the effect of TAC and AAV-PERM1 on mitochondrial energetics. Pressure overload markedly impaired both basal and maximal mitochondrial respiration in AAV-GFP hearts under both substrate conditions, as evidenced by significant reductions in non-phosphorylating oxygen flux (*J*O_2_) and maximal respiration (*J*O_2_ max; ΔG_ATP_-12.94), respectively (Fig. 2A–B, F–G). In contrast, AAV-PERM1 preserved mitochondrial respiratory capacity during pressure overload. Pyruvate/malate-supported respiration remained elevated above sham values, whereas octanoyl-carnitine/malate-supported respiration was restored to levels comparable to sham controls (Fig. 2A–B, F–G), suggesting that PERM1 preferentially preserves pyruvate-supported over fatty acid-supported respiration. Similarly, TAC reduced *J*O_2_ across the entire range of ATP free energies (ΔG_ATP_ =−14.45 to −13.38), whereas AAV-PERM1 maintained higher *J*O_2_ with pyruvate/malate and restored *J*O_2_ to sham levels with octanoyl-carnitine/malate throughout this energetic range (Fig. 2C,H).

Mitochondrial conductance, calculated from the relationship between *J*O_2_ and ATP free energy (ΔG_ATP_), provides an index of the efficiency with which OXPHOS couples ATP demand to respiratory flux. Pressure overload significantly reduced mitochondrial conductance under both substrate conditions (Fig. 2D,I), indicating impaired energetic transduction and a reduced capacity to match respiratory flux with ATP production. In contrast, AAV-PERM1 preserved mitochondrial conductance at sham levels with both substrates, indicating maintenance of efficient coupling between OXPHOS and ATP production during pressure overload (Fig. 2D,I).

Because pathological stress in heart failure is associated with increased oxidative stress and enhanced mitochondrial electron leak from the electron transport chain (ETC)^17^, we simultaneously measured H_2_O_2_ flux (*J*H_2_O_2_) in the same mitochondrial preparations used for *J*O_2_ analysis. Absolute *J*H_2_O_2_ production under non-phosphorylating conditions was not altered by either TAC or AAV-PERM1 (**Fig. S2A,E**). However, the relative electron leak (*J*H_2_O_2_/*J*O_2_) was significantly increased by TAC with pyruvate as a substrate and was completely normalized to sham levels by AAV-PERM1 (Fig. 2E). A similar trend was observed with octanoyl-carnitine, although neither TAC nor AAV-PERM1 significantly altered the percentage of electron leak (Fig. 2H).

These results show that AAV-PERM1 preserves mitochondrial OXPHOS capacity and energetic coupling during pressure overload. Although absolute mitochondrial *J*H_2_O_2_ was unchanged, PERM1 reduced electron leak and improved conductance, indicating more efficient electron transfer through the respiratory chain. Thus, the preservation of systolic function by AAV-PERM1 during pressure overload (Fig. 1) was accompanied by maintained mitochondrial function with both substrates, with preferential enhancement of pyruvate-supported respiration.

### AAV-PERM1 prevents pathological mitochondrial remodeling during pressure overload.

**Figure 3.**
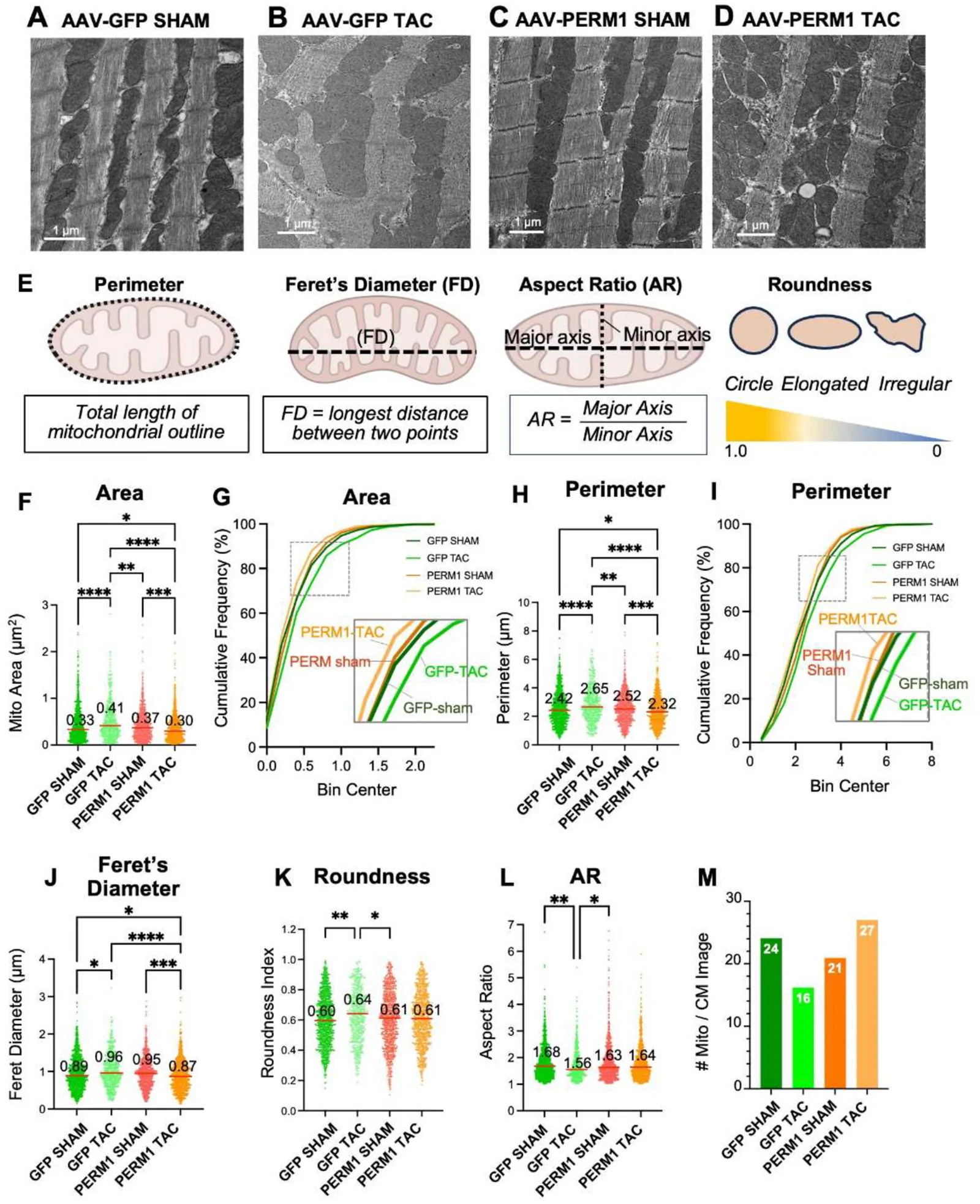
PERM1 promotes adaptive mitochondrial remodeling during pressure-overload. **A-D,** Representative transmission electron microscopy images of cardiac tissue from AAV-GFP sham **(A)**, AAV-GFP TAC **(B)**, AAV-PERM1 sham **(C)**, and AAV-PERM1 TAC **(D)** hearts. **E,** Schematic illustration of the four mitochondrial morphometric parameters used for quantitative analysis: perimeter (total length of the mitochondrial outline), Feret’s diameter (longest distance between two points on the mitochondrial boundary), aspect ratio (AR; ratio of major to minor axis), and roundness index (values approaching 1 indicate a perfect circle). **F-L,** Ultrastructure measurements including mitochondrial area (μm^2^) **(F-G)**, perimeter (μm) **(H-I)**, Feret’s diameter (μm) **(J)**, roundness index **(K)**, and aspect ratio **(L)** across all four groups. Statistical analyses were performed using Kruskal–Wallis with Dunn’s multiple-comparisons pairwise post hoc tests. *p < 0.05, **p < 0.01, ***p < 0.001, ****p < 0.0001. **(M)** Quantification of mitochondrial number per cardiomyocyte cross-sectional image across all four groups.

The effects of TAC and AAV-PERM1 on mitochondrial morphology was assessed by transmission electron microscopy. Quantitative analysis showed that pressure overload significantly increased the area, perimeter, Feret’s diameter, and roundness of interfibrillar mitochondria (IFM), all of which were attenuated by AAV-PERM1 treatment (Fig. 3A–K). Cumulative frequency analyses of IFM area and perimeter further demonstrated an increased probability of encountering enlarged, less elongated mitochondria after TAC, whereas AAV-PERM1 preserved a population of smaller, more elongated and compact mitochondria (Fig. 3G,I). Consistent with these changes, the aspect ratio (AR), an indicator of mitochondrial elongation, was significantly reduced in GFP-TAC hearts compared with GFP-sham (1.68 vs. 1.56 for GFP-sham vs. GFP-TAC, p<0.05), indicating a shift toward a less elongated morphology. This reduction was prevented by AAV-PERM1, with AR values in PERM1-TAC hearts remaining comparable to those in GFP-sham hearts (1.68 vs. 1.64 for GFP-sham vs. PERM1-TAC, p>0.05). In addition, TAC reduced mitochondrial abundance, while AAV-PERM1 maintained mitochondrial number at levels comparable to sham controls (Fig. 3M). These results indicate that PERM1 preserves mitochondrial morphology during pressure overload.

### AAV-PERM1 preserves mitochondrial biogenesis independently of mitochondrial dynamics.

**Figure 4.**
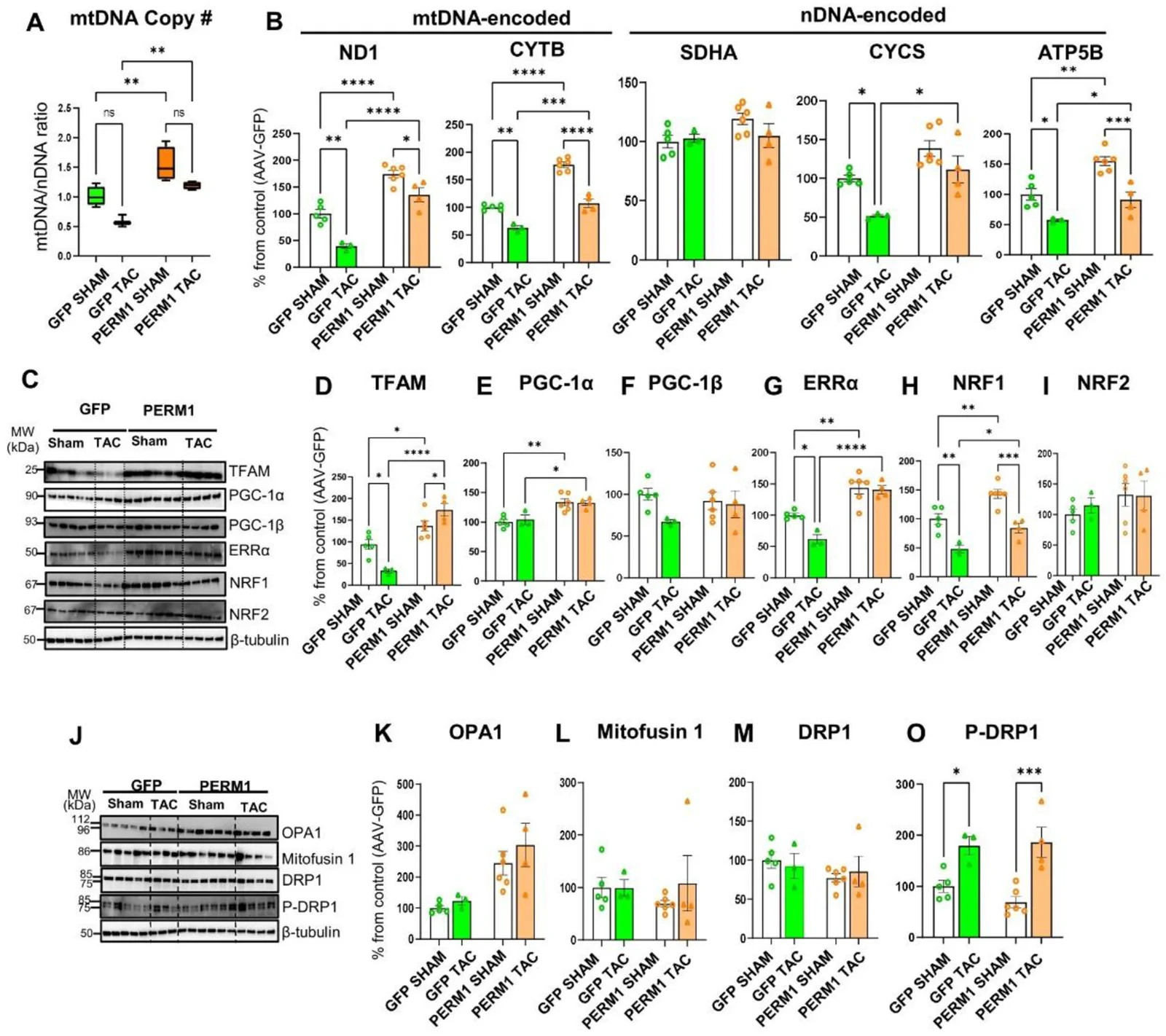
AAV-PERM1 promotes mitochondrial biogenesis independently of mitochondrial dynamics and mitophagy. **A,** TAC reduced mitochondrial DNA (mtDNA) copy number, which was completely prevented by AAV-PERM1. **B,** Protein expression of mtDNA-encoded and nuclear DNA (nDNA)-encoded OXPHOS proteins. **C-I,** Western blot analysis of transcription factors and co-factors involved in the regulation of mitochondrial biogenesis. **J-O,** Western blot analysis of proteins involved in mitochondrial dynamics and mitophagy. Data are presented as mean ± SEM. *p < 0.05, **p < 0.01, ***p < 0.001, ****p < 0.0001. Statistical analyses were performed using two-way ANOVA followed by Šidák post hoc pairwise comparisons test.

Because mitochondrial biogenesis is impaired in the failing human heart^18^, we next examined mitochondrial biogenesis and contents by quantifying mitochondrial DNA (mtDNA) copy number. TAC caused an approximately 50% reduction in mtDNA (normalized to nuclear DNA [nDNA]) in GFP-treated hearts, whereas AAV-PERM1 significantly increased mtDNA abundance under basal conditions and completely prevented mtDNA depletion during pressure overload (Fig. 4A). Preserved mtDNA was accompanied by increased abundance of both mtDNA-encoded (ND1 and CYTB) and nDNA-encoded OXPHOS proteins (CYCS and ATP5B), with a greater effect on mtDNA-encoded OXPHOS proteins (Fig. 4B). Interestingly, the preservation of these OXPHOS proteins by AAV-PERM1 during pressure overload was observed at the protein level, but not at the mRNA level (Fig. 4B, **Fig. S3**). Of note, neither TAC nor AAV-PERM1 altered the protein expression level of SDHA, a nDNA-encoded Complex II subunit (Fig. 4B).

Consistent with enhanced mitochondrial biogenesis, AAV-PERM1 markedly increased TFAM abundance, a key transcription factor for mtDNA replication, and upregulated PGC-1α, ERRα, and NRF1, the well-known transcription (co)factors that control mitochondrial biogenesis, in TAC hearts, while PGC-1α and NRF2 were unchanged by AAV-PERM1 (Fig. 4C–I). In contrast, proteins involved in mitochondrial dynamics and mitophagy were unaffected by AAV-PERM1 (Fig. 4J–O). There was no significant change in the protein expression levels of proteins involved in the regulation of mitochondrial fusion and fission (OPA1, MFN1, and DRP1) in either TAC or AAV-PERM-treated hearts (Fig. 4J–M). Notably, DRP1 phosphorylation was elevated by TAC in both AAV-GFP and AAV-PERM1 groups to a similar extent (Fig. 4J, O), indicating DRP1 activation occurred in response to pressure overload and was not further enhanced by AAV-PERM1.

Together with the preserved mitochondrial respiratory function and morphology (Fig. 2–3), these results indicate that AAV-PERM1 maintains mitochondrial quality and quantity during pressure overload primarily through preservation of mitochondrial biogenesis rather than modulation of mitochondrial dynamics or mitophagy.

### Metabolomics analysis reveals preservation of metabolic homeostasis by AAV-PERM1 during pressure overload.

To determine whether preserved mitochondrial bioenergetics by AAV-PERM1 altered the cardiac metabolome during pressure overload, we performed untargeted metabolomics on cardiac tissue from AAV-GFP and AAV-PERM1 mice subjected to sham or TAC surgery (8 weeks post-surgery, **Dataset 1**). Principal component analysis (PCA) revealed a significant shift in the metabolomics profile of GFP-TAC hearts compared with GFP-sham controls (**Fig. S4A**), indicating global metabolic remodeling induced by pressure overload. Integrated pathway enrichment and topology analysis using MetaboAnalyst 6.0 identified alterations in ketone body metabolism, the tricarboxylic acid (TCA) cycle, carnitine synthesis, and fatty acid and pyruvate metabolism (**Fig. S4B-C**). In contrast, PERM1-TAC hearts exhibited minimal separation from PERM1-sham hearts by PCA, with only modest changes in linoleic acid metabolism and amino acid metabolism (**Fig. S4D-E**). Direct comparison between GFP-TAC and PERM1-TAC hearts demonstrated distinct metabolic profiles (**Fig. S4F**). Notably, TAC-induced accumulation of spermidine and TCA cycle intermediates, including succinate, fumarate, and malate, was normalized by AAV-PERM1 (**Fig. S4G-H**). Collectively, these metabolomic analyses indicate that pressure overload disrupts multiple metabolic pathways linked to oxidative metabolism, whereas AAV-PERM1 partially restores metabolic homeostasis under pressure overload conditions.

### OXPHOS and fatty acid oxidation (FAO) genes remain suppressed in PERM1-treated hearts.

**Figure 5.**
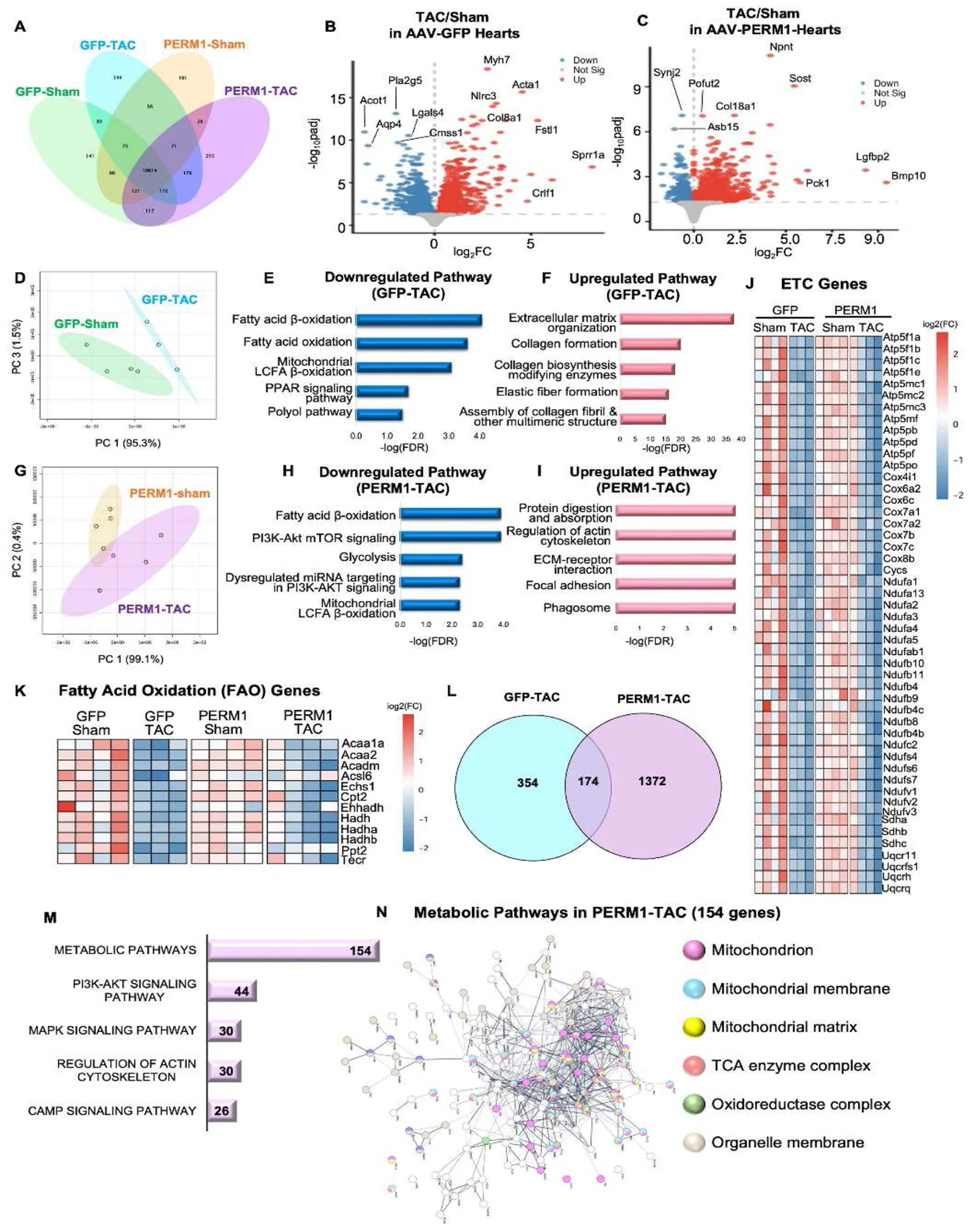
OXPHOS and fatty acid oxidation genes remain suppressed in PERM1-TAC hearts. RNA-seq analysis was performed on GFP-sham, GFP-TAC, PERM1-sham, and PERM1-TAC hearts. **A,** Venn diagram showing the overlap of expressed transcripts among GFP-sham, GFP-TAC, PERM1-Sham, and PERM1-TAC hearts. **B-C,** Volcano plot of differentially expressed genes in GFP-TAC hearts (**B**) and PERM1- TAC hearts (**C**) relative to their respective sham controls. Red dots indicate significantly upregulated genes, blue dots indicate significantly downregulated genes, and gray dots indicate genes that were not significantly altered (adjusted p<0.05). **D, G,** Principal component analysis (PCA) of RNA-seq data from GFP-TAC vs. GFP-sham hearts (**D**) and PERM1-TAC vs. PERM1-sham hearts (**G**). **E-F,** Wikipathways enrichment analysis of significantly downregulated (**E**) and upregulated (**F**) genes in GFP-TAC hearts (adjusted p<0.05, Datasets 1-2). **H-I,** Wikipathways enrichment analysis of significantly downregulated (**H**) and upregulated (**I**) genes in PERM1-TAC hearts (adjusted p<0.05, Datasets 3-4). **J-K,** Heatmaps showing TAC-induced downregulation of oxidative phosphorylation (OXPHOS) and fatty acid oxidation (FAO) genes, which maintained suppressed in AAV-PERM1-treated hearts. **L,** Venn diagram showing common and unique differentially expressed genes in GFP-TAC and PERM1-TAC hearts relative to their respective sham controls. **M,** KEGG pathway enrichment analysis of the 1,372 genes uniquely regulated by AAV-PERM1 during pressure overload, compared with GFP-TAC/sham hearts. Non-relevant pathways in the heart were excluded (highlighted in gray in Dataset 6), **N,** Gene ontology analysis of cellular components associated with the 154 genes within the metabolic pathways identified in panel M.

To determine whether the cardioprotective effects of AAV-PERM1 are mediated by transcriptional remodeling, we performed RNA sequencing of left ventricle tissue from GFP-sham, GFP-TAC, PERM1-sham, and PERM1-TAC hearts. A Venn diagram of expressed genes based on FPKM normalized values revealed substantial overlap among the four groups, indicating that a similar set of genes was detected in each condition (Fig. 5A). Pressure overload induced profound transcriptional reprogramming. PCA revealed a distinct transcriptomic profile of GFP-TAC hearts compared with GFP-sham hearts, and the volcano plot identified robust upregulation of genes associated with pathological cardiac remodeling, including *Myh7*, *Acta1*, *Col8a1*, and *Fstl1*, whereas the most strongly downregulated genes included those involved in fatty acid metabolism (*Acot1* and *Pla2g5*), membrane transport (*Aqp4*), and cell adhesion (*Lgals*) (Fig. 5B,D). Pathway enrichment analysis of 538 differentially expressed genes in GFP-TAC hearts revealed downregulation of FAO-related genes and upregulation of extracellular matrix organization and fibrotic pathways (Fig. 5E–F, **Datasets 2-3**). Unexpectedly, AAV-PERM1 alone did not alter the transcriptomic profile, as evidenced by the lack of separation between AAV-GFP-sham vs. AAV-PERM1-sham hearts in the PCA plot (**Fig. S5A**), and PERM1-TAC hearts remained transcriptionally distinct from PERM1-sham hearts (497 upregulated and 1,150 downregulated genes, Fig. 5C,G) and continued to exhibit suppression of FAO and long-chain fatty acid (LCFA) oxidation pathways (Fig. 5H; **Datasets 4-5**). Consistently, heatmap analysis showed persistent downregulation of genes encoding both OXPHOS and FAO proteins, including ATP synthase subunits, cytochrome c oxidase subunits, and NADH dehydrogenase subunits (Fig. 5J–K).

To identify shared and PERM1-specific transcriptional responses to pressure overload, we generated a Venn diagram comparing differentially expressed genes in GFP-TAC and PERM1-TAC hearts relative to their sham controls. This analysis showed that a shared pressure overload response includes extracellular matrix remodeling, branched-chain amino acid (BCAA) degradation, PI3K-Akt signaling, and fatty acid degradation pathways (**Fig. S5B; Dataset 6**). In contrast, genes uniquely regulated by AAV-PERM1 (1372 genes) were enriched for metabolic pathways and were predominantly localized to mitochondrial membrane, matrix, and mitochondrial compartments (Fig. 5M–N; **Datasets 7-8**).

These results indicate that the cardioprotective effects of PERM1 are not mediated by restoration of the pressure overload-induced transcriptional suppression of OXPHOS and FAO pathways. Instead, PERM1 promotes a distinct mitochondrial gene program associated with mitochondrial organization and protein homeostasis.

### AAV-PERM1 localizes to the mitochondria-sarcomere interface and associates with ribosomal proteins.

**Figure 6.**
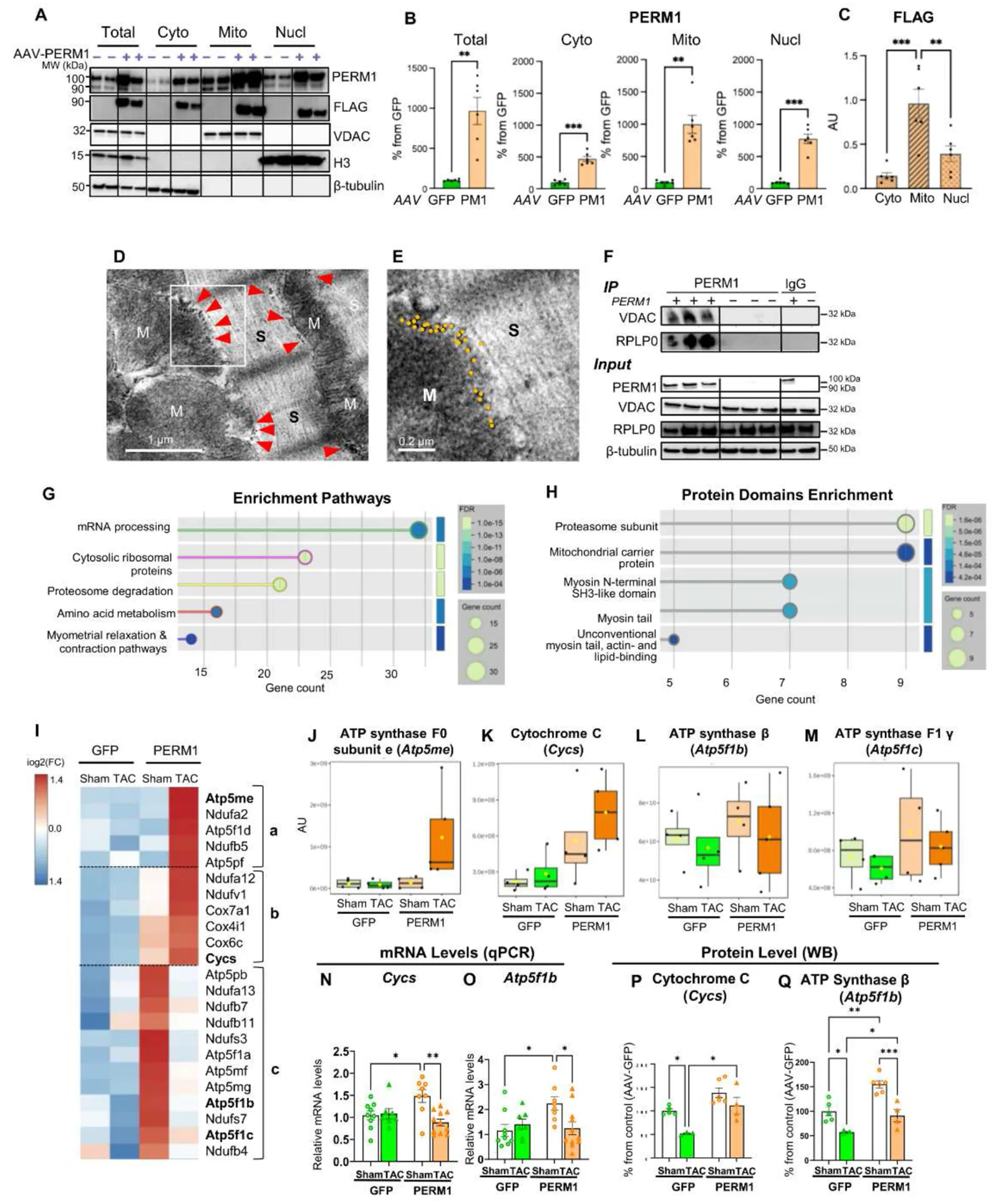
AAV-PERM1 localizes predominantly to mitochondria and associates with proteins involved in translation. **A-C,** Subcellular fractionation analysis of cardiac tissue. Endogenous PERM1 was detected in cytosol (Cyto), mitochondria (Mito), and nucleus (Nucl) fractions, with significantly increased PERM1 protein levels in all three fractions following AAV-PERM1 treatment. Fractions were validated using antibodies against β-tubulin, VDAC, and histone H3, respectively. Exogenous PERM1, detected using an anti-FLAG antibody, was also present in all three fractions, with the highest levels observed in mitochondria (**C**). n=6 per group. **D-E,** Representative immunogold electron microscopy images of AAV-PERM1-treated hearts showing the distribution of gold particles at interfibrillar mitochondria (*M*) adjacent to sarcomeres (*S*). Arrowheads indicate clusters of PERM1 localization. The white square in D is shown at higher magnification in **E**, with gold particles indicated by gold dots. **F,** Co-immunoprecipitation analysis confirming the interaction of PERM1 with VDAC (mitochondrial outer membrane protein) and RPLP0 (60S ribosomal subunit protein). **G-H,** Enrichment analysis of the PERM1 interactome. **I,** Heatmap showing proteomic changes in selected electron transport chain (ETC) subunits. ***Group a***: ETC proteins upregulated by AAV-PERM1 in TAC hearts; ***Group b***: ETC proteins upregulated by AAV-PERM1 in sham hearts and further increased during pressure overload; ***Group c***: ETC proteins upregulated by AAV-PERM1 in sham hearts and maintained at levels comparable to AAV-GFP sham controls during pressure overload. **J-M,** Box plots showing TAC-induced reductions or no significant changes in *Atp5me; Cycs; Atp5f1b; Atp5f1c* expression (highlighted in bold in Panel I), all which were increased in AAV-PERM1 TAC hearts. **N-O,** qPCR analysis showing minimal changes in *Cycs* (cytochrome C) and *Atp5f1b* (ATP synthase F1 subunit beta) mRNA following TAC and AAV-PERM1 treatment. **P-Q,** Western blotting confirming AAV-PERM1-induced changes in CYCS and ATP5F1B protein expression. The same blots shown in Figure 4B are included for comparison with corresponding mRNA data. Statistical analyses for Panel B used a paired t-test; Panel C, one-way ANOVA with Tukey’s post hoc test; Panels J-Q, two-way ANOVA with Šídák’s post hoc pairwise comparisons test. *: p < 0.05, **: p < 0.01, ****: p < 0.0001; data are presented as mean ± SEM.

Because AAV-PERM1 preserved mitochondrial bioenergetics (Fig. 2) without restoring OXPHOS or FAO gene expression (Fig. 5), we investigated whether PERM1 regulates mitochondrial function through a post-transcriptional mechanism. We first examined the subcellular localization of PERM1 in AAV-PERM1-treated hearts. Consistent with previous studies^12,19^, subcellular fractionation of ventricular tissue showed endogenous PERM1 expression to be present in the nuclear, cytosolic and mitochondrial fractions (Fig. 6A–B). More importantly, the PERM1 abundancy was significantly increased all in the mitochondrial, cytosolic and nuclear fractions (Fig. 6A–B). Similarly, exogenous FLAG-tagged PERM1 was enriched in the mitochondrial fractions, with lower abundance in the cytosol and nuclear fractions (Fig. 6C). Furthermore, immunogold electron microscopy localized PERM1 to the outer surface of interfibrillar mitochondria adjacent to sarcomeres (Fig. 6D–E). Co-IP further showed an interaction between PERM1 and the mitochondrial outer membrane protein VDAC (Fig. 6F), indicating localization of PERM1 to the mitochondrial outer membrane.

To gain insight into PERM1 function, we performed an unbiased analysis of a previously established PERM1 interactome^19^. Gene ontology analysis revealed enrichment of proteins involved in mRNA processing, proteasomal pathways, and, unexpectedly, cytosolic ribosomal proteins, including 18 proteins of the 60S and 10 proteins of the 40S ribosomal subunits (Fig. 6G, **Dataset 9**). The interaction between PERM1 and RPLP0, a core ribosomal protein, was validated by Co-IP (Fig. 6G). Protein domain enrichment further identified mitochondrial carrier proteins and myosin proteins (Fig. 6H, **Dataset 10**), consistent with the localization of PERM1 at the mitochondria-sarcomere interface (Fig. 6D–E). Given the absence of transcriptional remodeling of OXPHOS genes (Fig. 5) and PERM1’s interaction with ribosomal proteins (Fig. 6F–G), we hypothesized that AAV-PERM1 regulates OXPHOS proteins at the translational level. To test this hypothesis, we performed proteomics analysis of ventricular tissue, which revealed that AAV-PERM1 increased the abundance of a subset of electron transport chain (ETC) proteins (Fig. 6I–M) despite persistent transcriptional suppression of OXPHOS genes (Fig. 5). These proteins were classified into three categories: 1) ETC proteins increased by AAV-PERM1 during pressure overload (***Group a***); 2) ETC proteins increased by AAV-PERM1 under sham conditions and further elevated following TAC (***Group b***); and 3) ETC proteins increased in AAV-PERM1-treated sham hearts and maintained at levels comparable levels to GFP-sham hearts during pressure overload (***Group c***) (Fig. 6I–M). This post-transcriptional regulation was further confirmed by integrated qPCR and Western blotting analyses, showing discordant mRNA and protein expression of *Cycs* (Cytochrome C) and *Atp5f1b* (ATP synthase β) (Fig. 6N–Q). Additionally, proteomics analysis showed that the collagen proteins COL6A1 and COL4A2 were increased by pressure overload in AAV-GFP TAC, whereas AAV-PERM1 suppressed their protein abundance without altering their mRNA expression (**Fig. S6**). These results are consistent with the reduced cardiac fibrosis observed in AAV-PERM1-treated TAC hearts (Fig. 1N–O). Notably, neither TAC nor AAV-PERM1 altered the protein expression of CPT1b and CPT2, which mediate long-chain fatty acid transport into mitochondria, or MCAD, which catalyzes medium-chain fatty acid oxidation (**Fig. S7**).

Together, these results identify PERM1 as a component of a mitochondria-sarcomere microdomain that interacts with cytosolic ribosomal proteins and promotes post-transcriptional remodeling of the mitochondrial proteome. Rather than restoring OXPHOS and FAO gene expression, PERM1 selectively preserves ETC protein abundance, providing a mechanistic basis for preserved mitochondrial respiratory capacity during pressure overload.

### PERM1 preserves mitochondrial protein homeostasis by limiting pathological O-GlcNAcylation.

**Figure 7.**
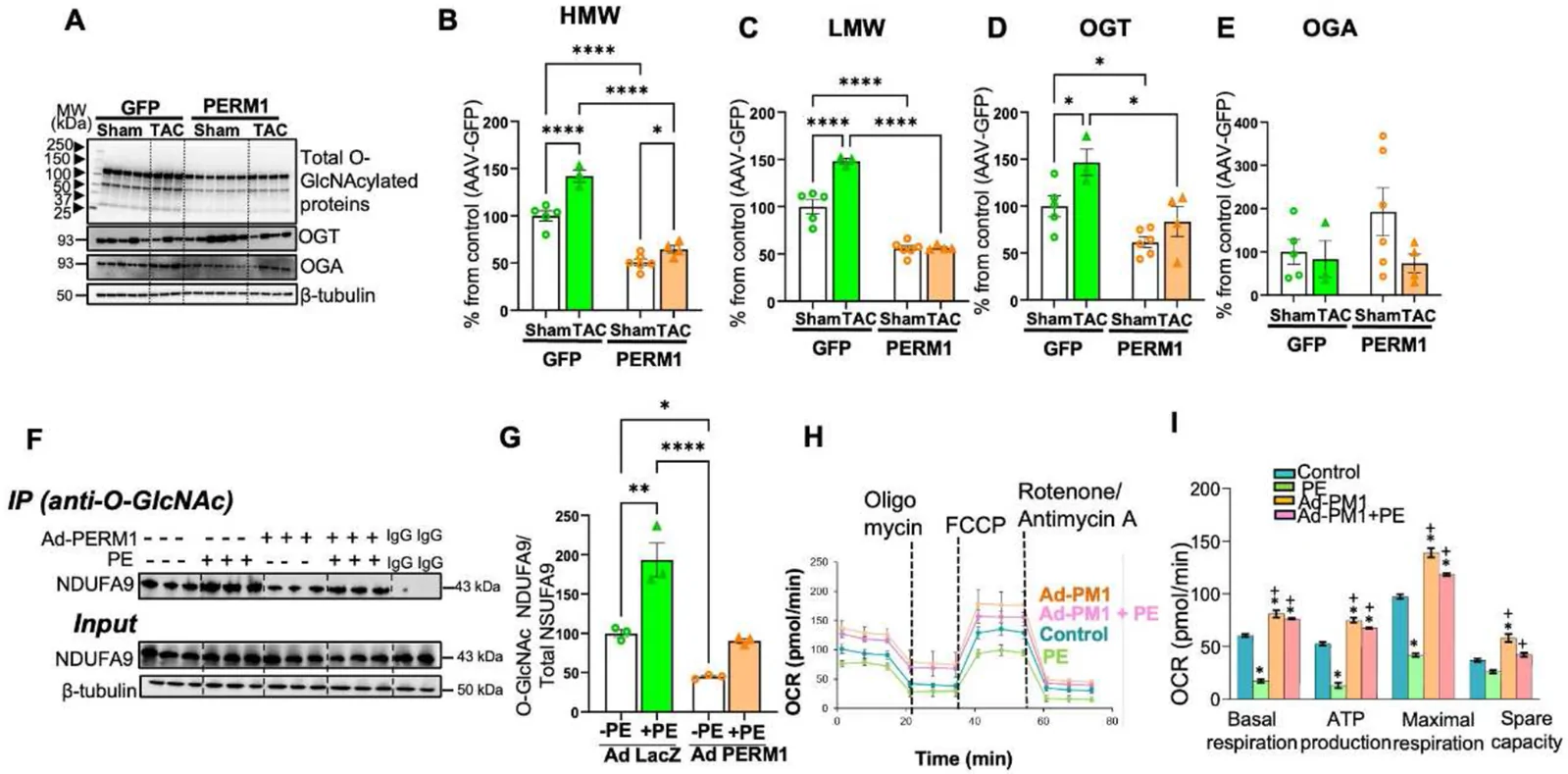
AAV-PERM1 suppresses TAC-induced pathological O-GlcNAcylation. **A-E,** Western blot analysis showing increased total O-GlcNAcylated proteins in both high-molecular-weight (HMW; ≥75 kDa) and low-molecular-weight (LMW; <75 kDa) fractions, together with increased OGT expression in GFP-TAC hearts compared with GFP-sham hearts, whereas OGA expression remained unchanged. In contrast, AAV-PERM1 reduced total O-GlcNAcylated proteins in both the HMW and LMW fractions and suppressed OGT expression under both basal and pressure overload conditions. O-GlcNAcylated proteins were quantified separately for HMW and LMW fractions (see Methods for details). **F-G,** Phenylephrine (PE)-induced hypertrophic stress increased O-GlcNAcylation of NDUFA9 (Complex I) in H9c2 cells, which was prevented by PERM1 overexpression. **H-I,** Seahorse Cell Mito Stress Test showing mitochondrial respiration capacity in H9c2 cells transduced with Ad-LacZ or Ad-PERM1 followed by PE treatment for 48h. PERM1 overexpression significantly increased mitochondrial respiration capacity in the presence of glucose and pyruvate as substrates (light green vs. orange). PE treatment significantly reduced mitochondrial respiration in control cells (light green vs. blue), whereas this reduction was prevented by Ad-PERM1, with respiration maintained at levels comparable to PERM1 overexpression alone (pink vs. orange). Statistical comparisons for **B-E and G** were performed using two-way ANOVA with Šídák post hoc pairwise test and statistical comparison for Panel **I** was performed using two-way ANOVA with Tukey post-hoc pairwise test. ****: p<0.0001, ***: p<0.001, *: p<0.05. Data are presented as mean ± SEM

Because PERM1 preserved mitochondrial bioenergetics without restoring metabolic transcripts (Fig. 5) and we previously showed that PERM1 regulates mitochondrial energetics through O-GlcNAcylation^20^, we next examined whether PERM1 also regulates protein homeostasis through O-GlcNAcylation. Consistent with previous studies^21,22^, pressure overload markedly increased total O-GlcNAcylated proteins and OGT expression (Fig. 7A–D). AAV-PERM1 completely prevented these changes, maintaining O-GlcNAcylation and OGT levels close to those of sham controls, whereas OGA expression remained unaffected by either TAC or AAV-PERM1 (Fig. 7A,E). Since excessive O-GlcNAcylation of mitochondrial ETC subunits including the Complex I subunit NDUFA9, contributes to impaired mitochondrial respiration^23^, we examined whether pathological O-GlcNAcylation of NDUFA9 occurs during hypertrophic stress and whether PERM1 suppresses this modification. H9c2 cells were transduced with either adenovirus LacZ (control) or adenovirus PERM1 (Ad-PERM1, PERM1 overexpression), followed by treatment with phenylephrine (PE). PE-mediated hypertrophic stress increased O-GlcNAcylation of NDUFA9, whereas PERM1 overexpression prevented this increase (Fig. 7F–G). To determine whether PERM1-dependent suppression of NDUFA9 O-GlcNAcylation preserves mitochondrial function, we assessed respiratory capacity using the Seahorse Cell Mito Stress Test in H9c2 cells transduced with Ad-LacZ (control) or Ad-PERM1 and subsequently treated with PE. Consistent with our previous studies^15,20^, PERM1 overexpression alone significantly increased basal, maximal, spare respiration and ATP production in the presence of glucose and pyruvate as substrates (all p<0.05, Ad-LacZ vs. Ad-PERM1, Fig. 7H–I). PE markedly reduced these parameters in control cells (Fig. 7H–I), coinciding with the increase in O-GlcNAcylated NDUFA9 observed under the same conditions (Fig. 7F–G). Notably, Ad-PERM1 completely abolished this PE-induced decline in basal, maximal, spare respiration and ATP production capacity, maintaining respiratory function at levels comparable to PERM1 overexpression alone (Fig. 7H–I). Together with the O-GlcNAcylation data, these functional results indicate that PERM1 preserves mitochondrial respiratory capacity during hypertrophic stress, at least in part, by limiting pathological O-GlcNAcylation of NDUFA9, linking PERM1-mediated protein homeostasis to preserved mitochondrial function.

### PERM1 links mitochondrial energetics to myofibrillar force generation.

**Figure 8.**
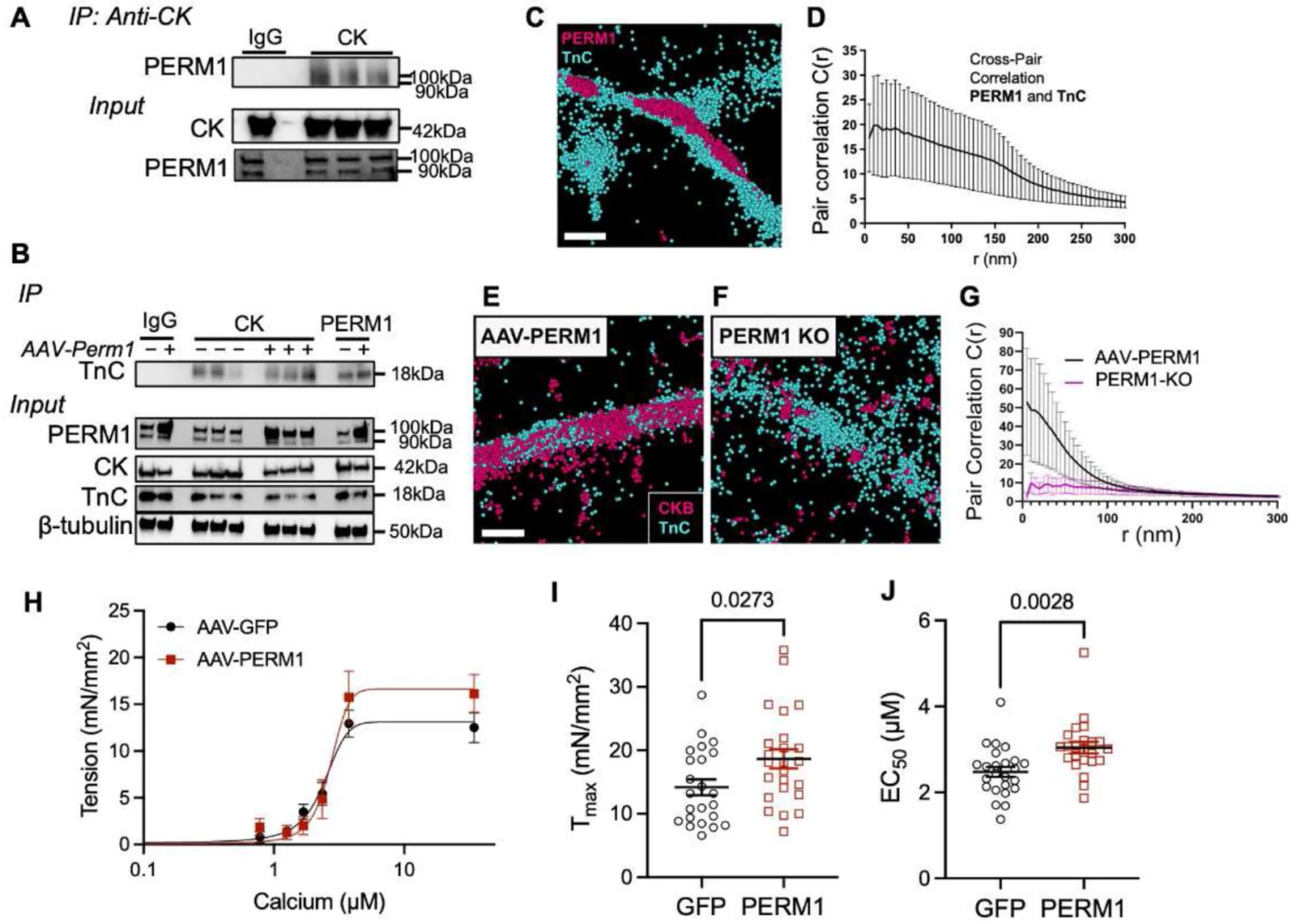
PERM1 interacts with creatine kinase (CK), promotes CK–troponin C (TnC) colocalization, and enhances myofibrillar force generation. **A-B,** Co-immunoprecipitation assays showing that PERM1 interacts with CK in WT hearts (**A**) and that TnC forms complexes with both CK and PERM1 in WT and AAV-PERM1-treated hearts (**B**). **C-D,** Super-resolution stochastic optical reconstruction microscopy (STORM) analysis of mouse cardiac tissue. STORM point-cloud localizations showing the spatial distribution of PERM1 and TnC in cardiac sections from AAV-PERM1-treated mice (**C**), with corresponding cross-pair correlation analysis demonstrating significant colocalization (**D**; n=4). **E-G,** STORM localizations of TnC and CK in AAV-PERM1 (**E**) and *Perm1*-KO (**F**) hearts. Cross-pair correlation analysis (**G**) revealed enhanced colocalization of TnC and CK in PERM1-overexpressing hearts compared with *Perm1*-KO hearts (n=4 per group). **H-J,** Tension–calcium relationship in skinned cardiomyocytes isolated from AAV-GFP- and AAV-PERM1-treated hearts (n=5 per group). Statistical comparisons for **I** was performed using an unpaired t test and statistical comparisons for **J** was performed using an unpaired t test with Welch’s correction.

Because AAV-PERM1 enhances cardiac contractility even in healthy hearts^13^, we hypothesized that PERM1 contributes to the coupling of mitochondrial ATP production to myofibrillar ATP utilization. Co-IP assays revealed an interaction between PERM1 and Creatine kinase (CK), a key mediator of phospho-transfer between mitochondria and the contractile apparatus (Fig. 8A). Furthermore, reciprocal pulldown assays using both anti-CK and anti-PERM1 antibodies demonstrated that troponin C (TnC) forms complexes with both CK and PERM1 in WT and AAV-PERM1-treated hearts (Fig. 8B). In contrast, neither CK protein abundance nor enzymatic activity was altered by loss- and gain-of-function of PERM1 in mice (**Fig. S8**), suggesting that PERM1 influences the organization rather than the abundance of the CK system. To determine whether these interactions affect contractile performance, we measured tension-calcium relationships in skinned cardiomyocytes. PERM1 overexpression significantly increased maximal calcium-activated tension (T_max_), while shifting the force-calcium relationship rightward, resulting in reduced calcium sensitivity (increased EC50) (Fig. 8H–J). Thus, PERM1 enhanced force generation without increasing calcium sensitivity.

Together, these results suggest that PERM1 contributes to mechano-energetic coupling by associating with a CK—troponin C complex and enhancing myofibrillar force generation, thereby linking mitochondrial energy metabolism to contractile performance.

## 3. Discussion

In this study, we demonstrate that AAV-mediated restoration of PERM1 prevents the development of pressure overload-induced HFrEF while preserving mitochondrial respiration, mitochondrial morphology, and contractile performance. Unexpectedly, these protective effects occurred despite persistent suppression of OXPHOS and FAO transcripts, revealing a mechanism distinct from canonical transcriptional metabolic reprogramming. Instead, our data identify PERM1 as a regulator of a sarcomere–mitochondria energetic microdomain that coordinates mitochondrial protein homeostasis and mechano-energetic coupling during pathological stress. These findings establish AAV-PERM1 as a promising gene therapy strategy for heart failure.

We recently showed that PERM1 expression is selectively restored in patients exhibiting myocardial recovery following LVAD implantation and that myocardial PERM1 abundance correlates with improved systolic function^14^. Whether this restoration represented a cause or consequence of recovery remained unresolved. The present study supports a causal role for PERM1 in maintaining cardiac function. Unlike PGC-1α, ERRα, and PPARα, whose sustained activation can aggravate cardiac dysfunction despite promoting mitochondrial gene expression^9,11,24,25^, PERM1 overexpression enhanced mitochondrial function and contractility without evidence of adverse remodeling, highlighting its therapeutic potential to fine-tune cardiac energetics and contractile function while preserving structural integrity.

Although PERM1 was originally characterized as a transcriptional coactivator of PGC-1α and ERRα, our findings indicate that its protective actions extend beyond transcriptional control. PERM1 localizes to the nucleus, cytosol, and mitochondria^12,19^, and recent work demonstrated that PERM1 associates with the MICOS-MIB complex and ankyrin B in skeletal muscle, linking mitochondria to the sarcolemma^26^. Constitutive cardiac PERM1 transgenic mice were reported to resist TAC-induced dysfunction^27^; however, most ETC and MICOS-MIB proteins were unaffected^27^, leaving unresolved whether PERM1 directly preserves mitochondrial bioenergetics during stress. Using high-resolution respirometry and simultaneous measurements of H_2_O_2_ emission and electron leak, we demonstrate for the first time that AAV-PERM1 preserves both pyruvate- and fatty acid-supported respiration and maintains mitochondrial energetic coupling efficiency during pressure overload. These functional benefits were accompanied by preservation of mitochondrial morphology, mtDNA abundance, and preferential maintenance of both mtDNA- and nDNA-encoded respiratory proteins together with increased TFAM, PGC-1α, and ERRα protein abundance, despite minimal effects on their transcripts. Previous studies showed that FAO genes were downregulated in PERM1-null hearts^19,28^ and that PERM1 overexpression in cultured cells promoted palmitate-supported oxidation^15,20^. In contrast, AAV-mediated PERM1 overexpression *in vivo* did not upregulate FAO genes at either mRNA or protein level. Instead, the enhanced fatty acid-supported respiration observed in sham and TAC hearts correlated more closely with increased ETC protein abundance than with changes in FAO enzymes and transporters.

These observations are particularly relevant given evidence that mitochondrial dysfunction in heart failure can occur independently of PGC-1α expression^18,29^. Human failing hearts exhibit depletion of mtDNA-encoded respiratory transcripts despite preserved PGC-1α abundance, suggesting that mitochondrial impairment is not simply a consequence of reduced transcriptional drive. Consistently, TAC did not reduce PGC-1α protein levels in our study, whereas AAV-PERM1 modestly increased PGC-1α abundance while preserving ETC protein expression through post-transcriptional rather than transcriptional mechanisms. Whether increased PGC-1α contributes to the enhanced bioenergetics in AAV-PERM1-treated hearts remains elusive, and this is difficult to address directly because PGC-1αdeletion alters the expression of multiple mitochondrial regulators, including PERM1, through transcriptional feedback mechanisms. Nevertheless, moderate cardiac PGC-1α overexpression fails to prevent pressure overload-induced dysfunction^24^ and can even accelerate disease progression^9^, thus, it is unlikely that increased PGC-1α alone accounts for the protection observed in this study. Instead, AAV-PERM1 preserved ETC proteins despite persistent suppression of OXPHOS transcripts and promoted only *modest* mitochondrial biogenesis, in contrast to the uncontrolled mitochondrial biogenesis and sarcomere disruption reported in PGC-1α transgenic hearts^8^. These results suggest that preservation of mitochondrial quality and mtDNA content, rather than activation of transcriptional programs alone, may represent a more effective therapeutic strategy for heart failure.

Our findings further point to post-transcriptional and post-translational mechanisms. PERM1 localized predominantly to the outer membrane of interfibrillar mitochondria adjacent to sarcomeres and associated with ribosomal proteins, while increasing mitochondrial ETC protein abundance without corresponding changes in transcript levels. In parallel, AAV-PERM1 suppressed pathological O-GlcNAcylation induced by pressure overload and prevented excessive O-GlcNAcylation of NDUFA9, a Complex I subunit. Previous studies showed that OGA overexpression restores Complex I activity and protects against pressure overload-induced dysfunction^23^, supporting a role for suppression of pathological O-GlcNAcylation in maintaining mitochondrial respiration. Together with studies by Lai and colleagues demonstrating that mitochondrial protein hyperacetylation contributes to respiratory defects in heart failure^30^, our findings suggest that maintenance of mitochondrial protein homeostasis, rather than transcriptional remodeling, represents a central mechanism underlying the cardioprotective effects of PERM1. Whether PERM1 also regulates mitochondrial acetylation warrants further investigation.

Beyond mitochondrial energetics, our results support a role for PERM1 in mechano-energetic coupling. Immunogold electron microscopy localized PERM1 to interfibrillar mitochondria adjacent to sarcomeres, whereas biochemical studies identified interactions with VDAC, creatine kinase, and troponin C. Although PERM1 did not alter total CK abundance or activity, it enhanced maximal myofibrillar force, supporting the concept that PERM1 can enhance both energy supply to the sarcomere and its intrinsic contractile function. This combination of effects ensures that the enhanced contractility is energetically balanced, an important dual function in the energetically compromised failing heart. The concomitant reduction in calcium sensitivity observed in skinned cardiomyocytes suggests preserved energetic and contractile efficiency during relaxation. Together, these results suggest that at the cellular level PERM1 finely tunes mechanics and energetics in concert to enhance systolic function while preserving diastolic function.

Several limitations should be acknowledged. Whether exogenous PERM1 fully recapitulates the functions of endogenous PERM1 remains unknown. Nonetheless, AAV-mediated PERM1 restoration prevented pathological remodeling and maintained mitochondrial function during pressure overload, highlighting PERM1 gene therapy as a promising and clinically translatable approach for heart failure. The present study does not establish whether PERM1 directly regulates translation or proteostasis, nor does it define the specific protein modifications responsible for improved mitochondrial function. Future studies employing spatial proteomics, ribosome profiling, and phospho-, acetyl-, and glycoproteomics will help elucidate how PERM1 coordinates mitochondrial protein homeostasis within the sarcomere–mitochondria interface.

In summary, AAV-mediated PERM1 restoration protects against pressure overload-induced heart failure by preserving mitochondrial protein homeostasis and coupling mitochondrial energetics to force generation. These findings identify PERM1 as a previously unrecognized regulator of the sarcomere–mitochondria energetic microdomain and support PERM1 gene therapy as a promising strategy for restoring both energetic and mechanical function in the failing heart.

## Experimental procedures

### Animals and tissue harvest

All animal experiments were approved by the Institutional Animal Care and Use Committee (IACUC) at Fralin Biomedical Research Institute (FBRI), Virginia Tech and were conducted according to the Guide for the Care and Use of Laboratory Animals. C57BL/6N female mice were purchased from Charles River. Animals were injected with AAV vectors carrying either PERM1 or GFP (control vector) at 8-12 weeks. Four weeks after AAV vector injection, animals were subjected to TAC surgery. Hearts were harvested from these animals 8 weeks after TAC surgery, immediately frozen in liquid nitrogen and stored at −80 °C until analysis. Male and female of *Perm1*-KO mice, previously generated^15,31^, were used at 10-12 weeks of age to evaluate the cardiac-specific effect of AAV-PERM1.

### AAV generation and administration in mice

AAV9 vectors encoding PERM1 (AAV9.CMV.mPerm1-FLAG.hGH) and GFP (AAV9.CMV.PI.EGFP.WPRE.Bgh) were obtained from the Penn Vector Core (RRID: SCR_022432; University of Pennsylvania) and Franklin Biolab (Pennsylvania, USA). AAV9.TNNT2.mPerm1-FLAG.hGH was generated by SignaGen laboratories (Frederick, MD, USA). AAV-PERM1 and AAV-GFP vectors were administered via retro-orbital injection into the venous sinus at a dose of 1 x 10^12^ genome copies (GC)/mouse, as previously performed^13^).

### Echocardiography

Mice were subjected to echocardiography at three time points: before AAV injection, 4 weeks after AAV injection (immediately before TAC), and 8 weeks after TAC (Table S1shows measurements at 8 weeks). All echocardiographic analysis were performed using Vevo 2100 (Visual Sonics) at FBRI. Mice were anesthetized in the induction chamber using 4% isoflurane in 100% O_2_ (2 L/min) and were maintained at 1.5 - 2.5% isoflurane and 0.8 L/min of O_2_ throughout the imaging studies. During the procedure, body temperature was maintained at 37.0 °C ± 1.0 °C, and heart rate was maintained in the range of 500 ± 50 beats per minute. Initially, two-dimensional (2D) imaging (B-mode) was done to obtain a view along the parasternal short axis followed by M-mode imaging to obtain high-resolution measurements of left ventricular diameter and wall dimensions in systole and diastole. These measurements were used to estimate the left ventricular ejection fraction (LVEF). Statistical analyses for Figure 1E–H were performed in Python using the pingouin library^32^. Analysis scripts were developed with AI assistance (Claude, Anthropic). The Python script used for computation of two-way ANOVA is presented in Dataset 11.

### Transverse Aortic Constriction Surgery.

Transverse aortic constriction (TAC) was performed 4 weeks after AAV administration as previously described^33^. Briefly, mice were anesthetized with isoflurane (2% in O_2_) and surgery was performed. A single dose of Ethiqa (buprenorphine sustained release) (0.15 mg/kg) was administered subcutaneously before surgery. Mice were placed in a supine position and an incision of about 0.5cm length was made along the neck to expose aortic arch overlaying the trachea. The aortic arch was visualized under a low-powered microscope, and a titanium microclip (Horizon) was placed at the aortic arch between the innominate artery and the left common carotid artery using a ligation clip applicator that was calibrated to the diameter of 32-gauge size needle. Mice were euthanized 8 weeks after TAC surgery (12 weeks after AAV administration), and heart tissue was collected for subsequent analyses. Sham-operated mice underwent identical surgical procedure except for placement of the microclip.

### Mitochondrial Respiratory Assays.

Mitochondria-enriched fractions isolated from cardiac muscle were used for these assays^16,33^. Ventricular tissue (~50-75 mg) was dissected for collection of a mitochondria-enriched fraction and placed immediately in 1mL of chilled mitochondria isolation buffer (MIB) [BSA (2 mg/mL), Sucrose (70 mM), Mannitol (210 mM), HEPES (5 mM), EGTA (1 mM), pH 7.1]. At 4°C, muscle was processed with a saw-tooth homogenizer for ~45 seconds. Homogenate underwent two centrifugations for 10 minutes at 4°C at 800 x g, then 9000 x g. Following the second spin the cytosolic fraction was collected from the supernatant and immediately frozen at −80°C. The final pellet was cleared of MIB by vacuum and resuspended in BSA-free MIB to be quantified by the Bradford method for normalization of protein loading for mitochondrial assays. Mitochondrial Respiratory Flux (*J*O_2_) Measurements. *Conceptual:* Mitochondrial respiration was assessed using the creatine kinase clamp method ^34,35^ for two separate substrate conditions: (1) malate/pyruvate (M/P) and (2) malate/octanoylcarnitine (M/Oct). This method employs creatine kinase (CK) to maintain the stoichiometric ratio of ATP:ADP in a range that reflects *in vivo* conditions, increasing the physiological relevance of our findings above traditional experiments that utilize a supraphysiological bolus of ADP to assess maximal respiration. Further, this method allows for calculation of conductance by titration of phosphocreatine (PCr), which reflects a tractable bioenergetic stress test mimicking the transition from lower demand (ΔG_ATP_) to higher demand. Calculations of ΔG_ATP_ were performed using a freely available github calculator provided here (https://dmpio.github.io/bioenergetic-calculators/ck_clamp/) with conditions of 37°C, 170 mM ionic strength, 5mM creatine, 10 mM phosphate, and pH 7.1 based on parameters referenced here (https://github.com/dmpio/bioenergetic-calculators/blob/master/jupyter_notebook/creatine-kinase-clamp.ipynb). Ranges of conductance included ΔG_ATP_ −12.94 kCal/mol for maximal respiration, −13.38, −13.71, −14.12, and −14.45 kCal/mol. Conductance of protons through the electron transport chain (reciprocal of resistance (1/R) is calculated as the slope over the linear range of *J*O_2_ vs. −13.38, −13.71, −14.12, and −14.45 based on Ohms law (I=V/R). *Procedural:* Oroboros O2k (Innsbruck, Austria) was maintained at 37°C and 500rpm in 0.5ml small volume chambers for each experiment. Chambers were calibrated with buffer D (BxD) [KMES (105 mM), KCl (30 mM), EGTA (1 mM), KH_2_PO_4_ (10 mM), MgCl_2_-6H2O (5 mM), 0.05%BSA, pH7.1, solubility factor 0.966] to assess R1 (air saturation, ~200 μM O_2_) and R0 (zero oxygen) with sodium hydrosulfide (Sigma S1256). Respiratory assessments of oxygen flux (*J*O_2_ (pmol/sec/mg)) were made with BxD supplemented with 5mM creatine monohydrate and 30μg of mitochondrial protein. Following addition of mitochondria substrate-mediated respiration (non-phosphorylating) was performed for two separate substrate conditions: (1) malate (2.5 mM) and pyruvate (5mM) and (2) malate (2.5 mM) and octanoyl carnitine (0.2 mM). Phosphorylating (maximal) respiration (ΔG_ATP_ −12.94) was assessed following the addition of ATP (5 mM), creatine kinase (20 mM), and PCr (1 mM). Cytochrome c (0.005 mM) was utilized to assess integrity with an *a priori* threshold of 15% increase in respiration, which did not require exclusion of any samples. Consecutive PCr titrations were then added at 1mM (ΔG_ATP_ −13.38 kCal/mol), 2 mM (ΔG_ATP_ −13.71 kCal/mol), 4 mM (ΔG_ATP_ −14.12 kCal/mol), and 6mM (ΔG_ATP_ −14.45 kCal/mol). Mitochondrial *J*H_2_O_2_ Emission. *Conceptual*: Evaluation of oxidative stress and reactive oxygen species production was assessed via the Amplex Ultra Red (AUR)/ horseradish peroxidase (HRP) Assay^36^ under the identical creatine kinase conditions described above with some modifications. The AUR assay detects the production of H_2_O_2_ as it reacts with HRP to produce resorufin that is detected fluorometrically. Addition of superoxide dismutase (SOD) aids in the inner-mitochondrial conversion of superoxide radicals to H_2_O_2_, which is then emitted outside of the mitochondria to be detected by AUR. Further, a portion of superoxide could be buffered by endogenous antioxidants and limit clear interpretation of ROS production within muscle mitochondria. To this end, we included auranofin, a thioredoxin reductase inhibitor, as this is a primary buffering system within cardiac and skeletal muscle ^37^. Auranofin has been further demonstrated to not impact respiration or conductance ^34^. A standard curve was performed to convert resorufin fluorescence to H_2_O_2_ picomoles and *J*H_2_O_2_ (pmol/min/mg). Electron leak provides for an estimation of oxygen consumed by ROS that does not contribute to *J*O_2_ expressed as a percentage (*J*H_2_O_2_/ *J*O_2_ *100) after converting *J*O_2_ to pmol/min/mg at corresponding experimental conditions. *Procedural:* Fluorescence was assessed via a fluorolog-QM (Horiba Scientific, Edison, NJ) outfitted with a 4-rotor turret system, temperature control, and stir bar capacity (Quantum Northwest, Liberty Lake, WA). Reactions occurred in a 600μl total volume with adapter cuvette at 37°C, 500 rpm, 565:600 ex/em conditions over 4 minutes for each reaction to reach a steady state for each *J*H_2_O_2_ calculation. Buffer D described from *J*O_2_ experiments was supplemented further with SOD (20 U/mL), HRP (1 U/mL), auranofin (0.001 mM), and AUR (0.02 mM) and collected for a background rate. 600μg of mitochondria was added followed by substrates separately assessed under the M/P and M/Oct conditions for non-phosphorylating respiration. Phosphorylating respiration was assessed following addition of ATP (5 mM), creatine kinase (20 U/mL), and PCr (1 mM). Conductance *J*H_2_O_2_ was assessed at ΔG_ATP_ −13.71 and −14.45 kCal/mol with 3 mM and 10 mM PCr additions, respectively.

**Table:** 

Reagent	Source	Product #	CAS#
**Sucrose**	Fischer	BP220	57-50-1
**Mannitol**	Sigma	M4125	69-65-8
**EGTA**	Sigma	E4378	67-42-5
**HEPES**	Gibco	15630-080	7365-45-9
**BSA**	Sigma	A6003	9048-46-8
Buffer D (pH 7.1)			
Reagent	Source	Product #	CAS#
**KMES**	Sigma	M0895	39946-25-3
**KCl**	Sigma	P4504	7447-40-7
**EGTA**	Sigma	E4378	67-42-5
**KH_2_PO_4_**	Sigma	P0662	7778-77-0
**MgCl2-6H2O**	Sigma	M2670	7791-18-6
**BSA**	Sigma	A6003	9048-46-8
*J*O_2_ Mitochondrial Respiration Flux			
Reagent	Source	Product #	CAS#
**Sodium Hydrosulfide**	Sigma	S1256	7775-14-6
**Potassium Pyruvate**	Combi-Blocks	QA-1116	4151-33-1
**Malate**	Sigma	M7397	97-67-6
**Octanoyl Carnitine**	Sigma	50892	25243-95-2
**Creatine Kinase from Rabbit muscle**	Sigma	10736988001	
**Phosphocreatine**	Sigma	P1937	108321-17-1
**ATP**	Sigma	A2383	102047-34-7
**Cytochrome C**	Sigma	C2506	9007-43-6
**Creatine Monohydrate**	Sigma	C3630	6020-87-7
*J*H_2_O_2_ Amplex Ultra Red Assay			
Reagent	Source	Product #	CAS#
**Amplex Ultra Red**	ThermoFischer	36006	
**Auranofin**	Sigma	A6733	
**Horseradish Peroxidase**	Sigma	P8375	9003-99-0
**Superoxide Dismutase**	Sigma	S9697	9054-89-1

### Cell Mito Stress Test.

Mitochondrial oxygen consumption rate (OCR) was assessed using the Agilent Seahorse XF Cell Mito Stress Test Kit (103015-100), as previously described^20,33^. Briefly, H9c2 cells (100k cells/well) were seeded in Seahorse XFe96 cell culture microplates and on reaching 70% confluency, were transduced with Ad-LacZ (control) or Ad-PERM1 (MOI 50) for 24 h, followed by treatment with or without 10 μM phenylephrine (PE; Sigma #P6126-5G) for 48 h. OCR was measured following sequential injection of oligomycin (1 μM), FCCP (4 μM), and rotenone/antimycin A (1 μM), per the manufacturer’s protocol to assess basal respiration, maximal respiration, ATP production and spare respiration capacity. OCR values were normalized to cell number.

### Mitochondrial DNA copy number measurements.

Mitochondrial copy number was measured as described in our previous publication^13^. Briefly, DNA was extracted from heart tissues using the DNeasy Blood and Tissue kit (QIAGEN #69506) according to the manufacturer’s instructions, and 50 ng DNA was used to measure the relative copy number of mitochondrial DNA (mtDNA) and nuclear DNA (nDNA; for normalization) by qPCR using Taqman primers for mitochondrial gene *Nd1* and nuclear gene *Rpl32* respectively. Taqman primers used were as follows: for ND1: mt-ND1_CDU63R2, Rpl32: Mm07306626_gH.

### Co-immunoprecipitation (Co-IP)

For *in vivo* Co-IP, freshly harvested heart tissue samples from wild-type (WT) and *Perm1*-KO mice were lysed in lysis buffer containing 0.5% Triton X-100, 150 mM NaCl, 50 mM Tris-HCl, 1 mM NaF, 0.1 mM Na_3_VO_4_, 1 mM dithiothreitol (DTT), 1 mM EDTA, 1 mM ethylene glycol tetraacetic acid (EGTA), and an EDTA-free protease inhibitor cocktail tablet. Lysates (750 μg) were incubated overnight with 2 μg of either anti-PERM1 antibody (#HPA031711) or anti-IgG antibody (control; R&D Systems #MAB002) on a rotator for immunoprecipitation. Samples were then incubated with Protein G Sepharose beads for 1 h 30 min on a rotator and centrifuged at 10,000 rpm for 2 min. The supernatant was aspirated, and the pellet was washed four times with lysis buffer. Sample buffer (10 μl; Bio-Rad #1610737, supplemented with 2-mercaptoethanol; Bio-Rad #1610710) was added to the washed pellet, and the resulting supernatant was collected for immunoblotting following centrifugation. Anti-VDAC (Abcam #ab14734) and anti-RPLP0 (Abcam #ab88872) antibodies were used for immunoblotting.

For *in vitro* Co-IP, H9c2 cells were plated in 15-cm culture dishes and at approximately 70% confluency, transduced with Ad-PERM1 or Ad-LacZ (control) at a MOI of 50 for 24 h, followed by treatment with or without 10 μM phenylephrine (PE; Sigma #P6126-5G) for an additional 48 h. Cells were then harvested in lysis buffer, and immunoprecipitation was performed by incubating lysates (750 μg) with 2 μg of anti-O-GlcNAc antibody (#RL2; Abcam #ab2739) or anti-IgG antibody (R&D Systems #MAB002). All subsequent steps were identical to those described above for *in vivo* Co-IP. Anti-NDUFA9 (Abcam #ab14713) antibody was used for immunoblotting.

### Trichrome staining.

The mouse hearts were embedded in the optimal cutting temperature (OCT) and cut into 10 μm sections. The sections were stained with Masson’s trichrome stain (Masson’s Trichrome for Connective Tissue, Electron Microscopy Sciences, Hatfield, PA; Cat# 26367) to evaluate cardiac fibrosis and imaged under transmitted light at 10x magnification. The blue-green area occupied by collagen fibers in the interstitial space was qualified using Image J software as described in detail in our previous publication^13^ and presented as a percent of the total area.

### Transmission electron microscopy imaging.

Left ventricular tissues were dissected, washed in PBS, and placed whole in 4% paraformaldehyde and 2.5% glutaraldehyde in PBS at room temperature. Then, rocked for 30 mins at room temperature and stored in 4°C until experiment day. Tissues were cut into pieces and washed with PBS twice with 10-minute intervals. Tissues were post-fixed in freshly prepared 1% osmium tetroxide containing 3-5 grains of potassium ferrocyanide in double-distilled water (ddH_2_O) for one hour. Tissues were then washed in ddH20 for 10 minutes, washed three times, followed by en bloc staining in 1% aqueous uranyl acetate for one hour at room temperature. Tissues were dehydrated through a graded ethanol series (50%, 70%, 90%, and 100% ethanol), 10 min twice at each step, and subsequently transitioned through 1:1 ethanol/propylene oxide for 10 minutes then 100% Propylene Oxide for 10 minutes twice, followed by an overnight incubation in 1:2 propylene oxide/epoxy resin. After three exchanges in 100% epoxy resin (two hours each), tissues were embedded in molds and baked at 60°C overnight. Tissue blocks were excised from the polymerized resin using a jeweler’s saw and mounted onto BEEM capsule supports using cyanoacrylate adhesive. Blocks were sectioned on a Leica UC7 ultramicrotome, and ultrathin sections (~60-90 nm) were collected onto 200-mesh formvar/carbon-coated copper grids. Sections were examined using a FEI Tecnai G2 Sprit Bio-Twin transmission electron microscope operated at 120 kV, and images were acquired digitally at 2900x magnification. Morphometric analysis. Morphometric analysis of mitochondria was performed using ImageJ/FIJI (NIH). All images were calibrated prior to measurement and only mitochondria with discernible boundaries were included in the analysis. Circularity and roundness were calculated as 4π x area/perimeter^2^ and 4 x are/(π x major axis^2^), respectively. Feret’s diameter represents the longest straight-line distance across a mitochondrion, measured between any two points on the outer boundary of individual mitochondria. Raw data generated from ImageJ were analyzed using Prism 9.

### Electron Microscopy with Immunogold Labeling.

Tissue samples were fixed in 0.05% glutaraldehyde and processed through an ethanol series at −20°C and embedded in Lowicryl K4M (EMS 14330). UV polymerization was achieved at −20°C using a wavelength of 360 nm for 48hrs. Ultrathin sections were then obtained at 70 nm using a Leica EM UC7 ultramicrotome and collected on Nickel grids. These sections were then incubated with anti-PERM1 primary antibody (1:100; HPA031711, Sigma-Aldrich, St. Louis, MO, USA) in 1% BSA and 1% Triton X-100 (EM Grade) overnight at 4°C, followed by incubation with 6-nm colloidal gold (H+L)-conjugated secondary antibody (1:50, AffiniPure^®^ 111-195-144) for 2 h at room temperature. Electron micrographs were acquired using a FEI Tecnai G2 Sprit Bio-Twin transmission electron microscope at 120 kV.

### Super-resolution STORM localization and analysis.

Tissue was fixed in 2% paraformaldehyde (PFA) at 4 °C for 20 min, followed by 3 washes with PBS for 5 min each. Tissue was blocked with 5 % normal donkey serum and 0.1 % Triton X-100 in 1x PBS for 1 h at room temperature. Primary antibody labeling was performed overnight at 4°C with rabbit anti-TnnC1 (1:100; ab137130, Abcam, Waltham, MA, USA) and mouse anti-Flag (1:100; F3165-2MG, Sigma-Aldrich, St. Louis, MO, USA) and anti-CKB (1:100; 66764-1-Ig, ProteinTech, Rosemont, IL, USA). Samples were washed six times (two quick PBS washes, two 10-min PBST (PBS containing with 0.05% tween 20) and two additional quick PBS washes) prior to secondary antibody labeling for 1 h at room temperature with donkey antibodies conjugated to Alexa Fluor 647 (Jackson ImmunoResearch, West Grove, PA, USA) or CF568 (Biotium, Hayward, CA, USA). Tissues were washed 6 times and kept in PBS at 4°C until ready for imaging. Stochastic optical reconstruction microscopy (STORM) was conducted with a Vutara SR-350 microscope (Bruker, Billerica, MA, USA). Tissues were imaged in 50 mM Tris-HCl, 10 mM NaCl, 10% (wt/vol) glucose buffer containing 20 mM mercaptoethylamine, 1% (vol/vol) 2-mercaptoethanol, 168 active units/ml glucose oxidase, and 1404 active units/ml catalase. A total of 2,500 frames were acquired for each probe, and 3D localizations were reconstructed in Vutara SRX software. Coordinates of localized molecules were used to calculate cross-correlation (cross-pair correlation) functions in the Vutara SRX software, and graphs were made from Prism 10.

### Skinned Myocyte Tension-Ca^2+^ Experiments.

Skinned cardiomyocyte tension-Ca^2+^ measurements were performed as previously described protocol^38^. Briefly, flash frozen left ventricular endomyocardial tissue was thawed directly into isolation buffer containing protease and phosphatase inhibitor cocktails (1:100, Fisher Scientific) and 10 mM DTT (Millipore Sigma). Sarcolemmal permeabilization was achieved by addition of 0.3% (v/v) Triton X-100 (Millipore Sigma) combined with brief mechanical disruption using three one-second homogenization pulses, followed by a 20-min incubation period on ice. Cellular debris and intact myocytes were then separated by low-speed centrifugation (120 × g, 2 min). The myocyte-enriched pellet was collected and reconstituted in isolation solution without Triton X-100. Myocytes were stored on ice prior to use. To assess myofibrillar function, single myocytes were selected under microscope and anchored at both ends to experimental pins using UV-sensitive adhesive (NOA 61, Thorlabs). One pin was connected to a force transducer (AE-801, Kronex) and the other pin was connected to a high-speed piezo length controller (Thorlabs). Myocytes were first perfused with a maximal calcium activating solution to elicit contraction, followed by 100% relaxing solution to facilitate relaxation. Tension was measured across a series of five intermediate calcium concentrations prepared by mixing activating and relaxing solutions. Sarcomere length was held constant at 2.1 μm throughout all trials, confirmed in real time by Fast Fourier Transform-based optical analysis, and all experiments were conducted at room temperature. Tension-calcium data were fit to the Hill equation: T = T_max_ × Ca^h^ / (EC^h^_50_ + Ca^h^) where Tmax denotes peak force-generating capacity, EC_50_ represents Ca^2+^ required to achieve 50% maximal force, and h is the Hill coefficient reflecting the degree of cooperative thin filament activation.

### RNA-Seq.

Ventricular tissue samples were processed, and RNA sequencing was performed commercially by Novogene (Sacramento, CA) using the Illumina NovaSeq X Plus platform (paired-end 150 bp sequencing). Raw count normalization was performed using DESeq2 (v1.46.0) in R (v4.4.3). To exclude low-count genes prior to DESeq2 normalization, only genes with at least 10 counts in one sample were retained. Differential gene expression analysis was performed using DESeq2 with Benjamini-Hochberg multiple comparison adjustment and apeglm (v1.28.0) shrinkage. An adjusted p-value (padj) < 0.05 and an |log_2_(FC)| > 1 was set as the threshold for differentially expressed genes (DEGs). Volcano plots displaying -log_10_(padj) and log_2_(FC) of DEGs were generated in R. *Pathway Enrichment Analysis.* Pathway enrichment analysis was performed on DEGs with a padj < 0.05 with STRING database (https://string-db.org). For individual KEGG pathway heat maps, normalized counts for all DEGs identified in the GFP-TAC versus GFP-sham comparison were extracted and a per-gene z-score was calculated across samples to visualize relative expression patterns.

### Western blotting analysis.

Ventricular tissue for Western blotting analysis was prepared and analyzed as we previously performed^13–15,19,20,33^. Briefly, tissue samples were lysed in lysis buffer containing 50 mM Tris (pH 7.4), 10 mM EDTA, 1% sodium dodecyl sulphate (SDS), and protease inhibitors that include sodium butyrate (NaB), phenylmethyl sulphonyl fluoride (PMSF), sodium vanadate (Na_3_VO_4_), sodium fluoride (NaF) and EDTA-free protease inhibitor cocktail tablet. The lysate was centrifuged at 13,000 g for 10 min at 4°C and the supernatant was subjected to SDS electrophoresis followed by western blotting using 0.2-μm nitrocellulose membrane (Bio-Rad#1620112). The protein levels of PERM1, TFAM, PGC-1α, PGC-1β,ERRα,PPARα,NRF1,NRF2, cytochrome C, ATP synthase β, CPT1b, CPT2, MCAD, OGT, OGA, OPA1,MFN1 were detected using rabbit antibodies (Sigma #HPA031711, Abcam #ab307302, Abcam #ab191838, Abcam #ab176328, Abcam #ab76228, Cayman product code #101710, Cell Signaling #D9K6P, Cell Signaling #D129C, Abcam #ab110325, Abcam #ab14730, Cell Signaling #E6M5M, Cell Signaling #E7D4W, Abcam #ab92461, Cell Signaling #24083, Abcam #ab124807, Abcam #ab56889-100 and Novus Biologicals #NBP2-34206) with 1:1,000 dilution followed by goat anti-rabbit secondary antibody (Jackson ImmunoResearch Laboratories, Inc. #711- 035-152) with 1:5,000 dilution. The protein levels of total O-GlcNAcylated proteins were detected using mouse antibody (RL2, Abcam #ab2739) with 1:1,000 dilution followed by anti-mouse secondary antibody from Abcam #ab6728 with 1:5000 dilution. The values of band intensities were normalized using ^®^-tubulin (Abcam, #ab6046). The protein expression levels of β-tubulin were consistent in all groups that were used in this study. For the quantification of O-GlcNAc blots (Fig. 7), the blots were divided into two portions: bands ≥75 kDa (High molecular weight, HMW) and bands <75 kDa (Low molecular weight, LMW). In the HMW portion, non-specific bands around 75 kDa, which resulted from IgG band staining, were excluded by re-immunoblotting samples with anti-O-GlcNAc antibody preincubated overnight with 50 mM of N-acetylglucosamine, an O-GlcNAc specific sugar. Both HMW and LMW bands were quantified separately.

### Global proteome profiling analyses.

Label-free proteomics analysis of ventricular tissue was performed as previously described^39^. Briefly, label-free quantitation (LFQ) intensities were transformed (log2). Proteins with less than at least two valid values were filtered out. For each protein, missing values within samples from the same comparison group were imputed using the k-nearest neighbors (KNN) algorithm if at least 60% of the values in that group were valid. Thereafter, the remaining missing values were imputed using the MinProb approach (width = 0.3, shift = 1.8). Unpaired *t* test comparisons across relevant conditions were performed with differentially expressed proteins considered statistically significant following 250-permutation based false discovery rate correction, at a level of 0.05. Heatmap and violin plots were generated from LFQ intensity values using Metaboanalyst.

### Gene expression analysis.

Real-time PCR was performed using Taqman primers as previously described^14,15,19,20,33^. Briefly, RNA was extracted using TRizol (Life Technologies), followed by ethanol extraction. Reverse transcription was performed from 1μg of RNA using QuantiTect Reverse Transcription Kit according to the manufacturer’s instruction (Qiagen). Taqman primers were purchased from ThermoFisher as follows: *Nppb*: Mm01255770_g1, *Col3a1*: Mm00802300_m1, *Col5a2*: Mm01254423_m1, *Nd1:* Mm04225274_s1, *Nd6:* Mm04225325_g1, *Sdha:* Mm01352366_m1, *Cycs:* Mm01621048_s1, *Atp5f1b:* Mm01160389_g1, *Hadhb:* Mm00695255_g1, *Ehhadh:* Mm00619685_m1, *Acaa2*: Mm00624282_m1. The Cq values from the genes were normalized using *Rpl32* (Mm07306626_gH).

### Metabolomic Analysis.

Untargeted metabolomic screening was performed using the Shimadzu GCMS-TQ8050 NX EI/CI/NCI mass spectrometry system, as described previously^19,33,40,41^. Briefly, freeze-clamped ventricle tissue samples (~12 mg) were homogenized in an extraction solution consisting of 90% methanol/water and containing d4-succinate and d4-myristic acid as internal standards. For plasma metabolomics, blood was collected using heparin at the time of terminal study and centrifuged at 1,500 x *g* for 10 minutes at 4°C. Ten microliters of plasma were mixed with 400 ìL of the same extraction solution. All samples were dried and derivatized sequentially with O-methoxylamine hydrochloride in pyridine, followed by N-Trimethylsilyl-N-methyl trifluoroacetamide prior to GC-MS analysis. Bioinformatic analysis was conducted using MetaboAnalyst 6.0, which included the generation of heat maps and pathway impact analysis (**Fig. S4**). Pathway analysis integrated p-values from pathway enrichment and visualized results where node color indicated statistical significance and node size reflected pathway impact values. Differences in metabolite abundance were evaluated using two-way ANOVA, with a p<0.05 considered statistically significant. Data are presented as mean ± SEM.

### Statistical analysis

The data are presented as the mean ± SEM and were analyzed using GraphPad Prism 10.0, except for the echocardiographic data in Figure 1E–H, which were analyzed in Python using the pingouin library as described above. Two-way ANOVA was followed by pairwise comparisons using Šídák’s test (Figures 1, 4, 6J–Q, and 7B–E,G), Holm-Šídák’s test (Figures 2, 3), Tukey’s test (Figure 7I; all pairwise comparisons), Kruskal-Wallis test with Dunn’s multiple comparisons as Post Hoc (Figure 3), one-way ANOVA with Tukey’s test (Figure 6C), or two-tailed Student’s t-test (Figure 6B, and Figure 8), as appropriate. The statistical test used for each analysis is also indicated in the corresponding figure legends. A value of p < 0.05 was considered statistically significant.

## Supplementary Material

This is a list of supplementary files associated with this preprint. Click to download.

- TableS1.Echo8wkTAC.xlsx
- TableS1.Echo8wkTAC.xlsx
- TableS1.Echo8wkTAC.xlsx
- TableS1.Echo8wkTAC.xlsx
- Dataset1GCMSMetabolomics.xlsx
- Dataset1GCMSMetabolomics.xlsx
- Dataset1GCMSMetabolomics.xlsx
- Dataset1GCMSMetabolomics.xlsx
- Dataset3RNAseqUpregulatedPathwayGFPTACFig5F.xlsx
- Dataset3RNAseqUpregulatedPathwayGFPTACFig5F.xlsx
- Dataset3RNAseqUpregulatedPathwayGFPTACFig5F.xlsx
- Dataset3RNAseqUpregulatedPathwayGFPTACFig5F.xlsx
- Dataset11echo2wayanovaClaude08Jun26.xlsx
- Dataset11echo2wayanovaClaude08Jun26.xlsx
- Dataset11echo2wayanovaClaude08Jun26.xlsx
- Dataset11echo2wayanovaClaude08Jun26.xlsx
- Dataset8RNAseqMetabolicpathways154genesFig5N.xlsx
- Dataset8RNAseqMetabolicpathways154genesFig5N.xlsx
- Dataset8RNAseqMetabolicpathways154genesFig5N.xlsx
- Dataset8RNAseqMetabolicpathways154genesFig5N.xlsx
- Dataset9InteractomeEnrichmentpathwaysFig6H.xlsx
- Dataset9InteractomeEnrichmentpathwaysFig6H.xlsx
- Dataset9InteractomeEnrichmentpathwaysFig6H.xlsx
- Dataset9InteractomeEnrichmentpathwaysFig6H.xlsx
- Dataset6RNAseqCommonlyAlteredPathinTAC174genesFigS5B.xlsx
- Dataset6RNAseqCommonlyAlteredPathinTAC174genesFigS5B.xlsx
- Dataset6RNAseqCommonlyAlteredPathinTAC174genesFigS5B.xlsx
- Dataset6RNAseqCommonlyAlteredPathinTAC174genesFigS5B.xlsx
- Dataset7RNAseqUniquielyAlteredPathinPERM1TAC1372genesFigS5B.xlsx
- Dataset7RNAseqUniquielyAlteredPathinPERM1TAC1372genesFigS5B.xlsx
- Dataset7RNAseqUniquielyAlteredPathinPERM1TAC1372genesFigS5B.xlsx
- Dataset7RNAseqUniquielyAlteredPathinPERM1TAC1372genesFigS5B.xlsx
- Dataset5RNAseqUpregulatedPathwayPERM1TACFig5I.xlsx
- Dataset5RNAseqUpregulatedPathwayPERM1TACFig5I.xlsx
- Dataset5RNAseqUpregulatedPathwayPERM1TACFig5I.xlsx
- Dataset5RNAseqUpregulatedPathwayPERM1TACFig5I.xlsx
- Dataset4RNAseqDownregulatedPathwayPERM1TACFig5H.xlsx
- Dataset4RNAseqDownregulatedPathwayPERM1TACFig5H.xlsx
- Dataset4RNAseqDownregulatedPathwayPERM1TACFig5H.xlsx
- Dataset4RNAseqDownregulatedPathwayPERM1TACFig5H.xlsx
- 20260825NatCommunSupplmaterialsAAVPerm1TACKS.pdf
- 20260825NatCommunSupplmaterialsAAVPerm1TACKS.pdf
- 20260825NatCommunSupplmaterialsAAVPerm1TACKS.pdf
- 20260825NatCommunSupplmaterialsAAVPerm1TACKS.pdf
- Dataset10InteractomeProteinDomainsEnrichmentPfamFig.6I.xlsx
- Dataset10InteractomeProteinDomainsEnrichmentPfamFig.6I.xlsx
- Dataset10InteractomeProteinDomainsEnrichmentPfamFig.6I.xlsx
- Dataset10InteractomeProteinDomainsEnrichmentPfamFig.6I.xlsx
- Dataset2RNAseqDownregulatedPathwayGFPTACFig5E.xlsx
- Dataset2RNAseqDownregulatedPathwayGFPTACFig5E.xlsx
- Dataset2RNAseqDownregulatedPathwayGFPTACFig5E.xlsx
- Dataset2RNAseqDownregulatedPathwayGFPTACFig5E.xlsx

## Data Availability

The data in support of the findings of this study may be found within the manuscript and in the associated supplementary files (datasets). RNA-seq raw have been deposited in the NCBI Gene Expression Omnibus (GEO) under accession number GSE338319. The data are currently private and will be made publicly available upon publication. A reviewer access token has been provided to the editor. Raw data (echocardiography images, original blots, original GC-MS runs files, and others) are available from authors upon a reasonable request.
